# Long non‐coding RNAs influence the transcriptome in pulmonary arterial hypertension: the role of *PAXIP1‐AS1*

**DOI:** 10.1002/path.5195

**Published:** 2019-01-16

**Authors:** Katharina Jandl, Helene Thekkekara Puthenparampil, Leigh M Marsh, Julia Hoffmann, Jochen Wilhelm, Christine Veith, Katharina Sinn, Walter Klepetko, Horst Olschewski, Andrea Olschewski, Matthias Brock, Grazyna Kwapiszewska

**Affiliations:** ^1^ Ludwig Boltzmann Institute for Lung Vascular Research Graz Austria; ^2^ Department of Internal Medicine Justus‐Liebig‐University Giessen, Universities of Giessen and Marburg Lung Center, German Center for Lung Research Giessen Germany; ^3^ Excellence Cluster Cardio‐Pulmonary System Justus‐Liebig‐University Giessen, Universities of Giessen and Marburg Lung Center, German Center for Lung Lung Research Giessen Germany; ^4^ Division of Thoracic Surgery, Department of Surgery Medical University of Vienna Vienna Austria; ^5^ Division of Pulmonology, Department of Internal Medicine Medical University of Graz Graz Austria; ^6^ Otto Loewi Research Center, Chair of Physiology Medical University of Graz Graz Austria; ^7^ Division of Pulmonology University Hospital Zürich, University of Zürich Zürich Switzerland

**Keywords:** lncRNA, vascular diseases, paxillin, pulmonary hypertension, vascular remodelling, pulmonary artery smooth muscle cells

## Abstract

In idiopathic pulmonary arterial hypertension (IPAH), global transcriptional changes induce a smooth muscle cell phenotype characterised by excessive proliferation, migration, and apoptosis resistance. Long non‐coding RNAs (lncRNAs) are key regulators of cellular function. Using a compartment‐specific transcriptional profiling approach, we sought to investigate the link between transcriptional reprogramming by lncRNAs and the maladaptive smooth muscle cell phenotype in IPAH. Transcriptional profiling of small remodelled arteries from 18 IPAH patients and 17 controls revealed global perturbations in metabolic, neuronal, proliferative, and immunological processes. We demonstrated an IPAH‐specific lncRNA expression profile and identified the lncRNA *PAXIP1‐AS1* as highly abundant. Comparative transcriptomic analysis and functional assays revealed an intrinsic role for *PAXIP1‐AS1* in orchestrating the hyperproliferative and migratory actions of IPAH smooth muscle cells. Further, we showed that *PAXIP1‐AS1* mechanistically interferes with the focal adhesion axis via regulation of expression and phosphorylation of its downstream target paxillin. Overall, we show that changes in the lncRNA transcriptome contribute to the disease‐specific transcriptional landscape in IPAH. Our results suggest that lncRNAs, such as *PAXIP1‐AS1*, can modulate smooth muscle cell function by affecting multiple IPAH‐specific transcriptional programmes. © 2018 The Authors. *The Journal of Pathology published* by John Wiley & Sons Ltd on behalf of Pathological Society of Great Britain and Ireland.

## Introduction

Pulmonary arterial hypertension (PAH) is a severe and progressive disease which ultimately leads to right heart failure 1. Within the vessel wall, multiple factors contribute to the increased pulmonary pressure, including cellular hyperplasia and extracellular matrix (ECM) deposition 2, 3. In the medial layer, smooth muscle cells (SMCs) show increased proliferation 4, invasive migratory capacity, and resistance to apoptosis 5. These functional alterations are accompanied by a reprogramming of cellular respiration 6 and glucose expenditure 7. PAH has a strong genetic predisposition, with several mutations contributing to the disease development 8. Loss‐of‐function mutations in *BMPR2* occur in over 70% of patients with familial PAH and in 25% of patients with the idiopathic form 9. However, *BMPR2* mutations show only 20% penetrance 10. Thus, it is apparent that additional regulatory mechanisms are involved and factors other than solely mutations in coding genes are required for disease development.

The human genome contains approximately equal numbers of coding and non‐coding genes (GENCODE Release, version 29). Despite being highly abundant, non‐coding genes are still under‐studied. The non‐coding transcriptome includes many regulatory transcripts, which can be roughly classified into small non‐coding RNAs (sncRNAs, < 200 nt), including the group of microRNAs (miRNAs), and long non‐coding RNAs (lncRNAs, > 200 nt) 11. Variations in disease complexity and penetrance of genetic variants are increasingly attributed to changes in gene expression of the non‐coding genome, rather than to changes within protein‐coding sequences 12.

In contrast to sncRNAs, which act via RNA binding, lncRNAs exert their regulatory effects, both at the transcriptional and at the post‐transcriptional levels, via interactions with DNA, chromatin, and other RNA species 13. In addition, lncRNAs can bind chromatin‐modifying proteins such as histone modifiers, and thus act as epigenetic modulators 14, 15. In this respect, lncRNAs often regulate fundamental cellular processes, such as proliferation or apoptosis 16, 17. Due to the tight transcriptional regulation of lncRNAs and their specific subcellular localisation, a ‘long non‐coding’ landscape can be generated, which is highly cell‐type, tissue, and disease‐specific 11, 18, 19.

In the field of pulmonary vascular remodelling, sncRNAs such as miRNAs have been intensively studied and constitute well‐recognised cellular control mechanisms 20, 21, 22, 23, 24. In contrast, lncRNAs are under‐investigated in this field. In the present study, we have characterised the gene expression profiles of coding and non‐coding genes from small pulmonary arteries obtained via laser‐capture microdissection (LCM) from IPAH and control lungs. We focus on the functional role of a highly deregulated lncRNA, *PAXIP1‐AS1*, in perpetuating the migratory and proliferative phenotype of IPAH pulmonary artery smooth muscle cells (PASMCs).

## Materials and methods

### Genome‐wide expression profiling

Briefly, whole genome expression profiling was performed on material obtained from small pulmonary arteries (50–500 μm in diameter, consisting of intima and media) isolated using LCM from IPAH patients and down‐sized non‐tumorous, non‐transplanted donor lungs [approved by the institutional ethics committee (976/2010)]. The patients' key clinical characteristics are listed in Table 1. A detailed description of the procedure is provided in the supplementary material, Supplementary materials and methods.

**Table 1:** Clinical characteristics of the donors and IPAH patients in this study

	Donors (*n* = 17)	IPAH (*n* = 18)	*P* value
Age, years	46 ± 12	32 ± 12	0.0016
Sex, M/F	8/9	4/14	0.1218
HR, beats/min		87.8 ± 9.7	
mPAP, mmHg		76.3 ± 22.1	
PAWP, mmHg		10.6 ± 4	
RA, mmHg			
CO, l/min		3.5 ± 1.6	
CI, l/min/m^2^		2.1 ± 0.9	
PO_2_, mmHg		66.2 ± 19.3	
PCO_2_, mmHg		31 ± 6.1	
PVR, dyn·s/cm^5^		1665.4 ± 799.7	
6MWD, m		298.7 ± 188.2	
NT‐proBNP, pg/ml		4061.9 ± 2578	
CRP, mg/l		0.56 ± 0.5	
Bilirubin, mg/dl		1.8 ± 2.1	
Uric acid, mg/dl		7 ± 1.7	

6MWD, 6‐min walking distance; CI, cardiac index; CO, cardiac output; CRP, C‐reactive protein; F, female; HR, heart rate; IPAH, idiopathic pulmonary arterial hypertension; M, male; mPAP, mean pulmonary arterial pressure; NT‐proBNP, NH_2_‐terminal pro‐brain natriuretic peptide; PAWP, pulmonary arterial wedge pressure; PCO_2_, partial pressure of carbon dioxide; PO_2_, partial pressure of oxygen; PVR, pulmonary vascular resistance; RA, right atrial pressure.

### Bioinformatic analysis

Several online tools (RegRNA 2.0, CPC, CPAT, Vienna RNA Websuite, BLAST, NetworkAnalyst) 25, 26, 27, 28, 29, 30, 31, 32 were used to explore the RNA sequence and the obtained expression profiling data.

### Cell culture, transfection, and functional assays

Human PASMCs were bought (Lonza, Basel, Switzerland) or isolated from pulmonary arteries from non‐transplanted donor lungs or IPAH lungs. siRNA‐ and GapmeR‐mediated knockdown was performed 48 h before performing the functional experiments, details of which are given in the supplementary material, Supplementary materials and methods.

### Immunofluorescence staining and western blot analysis

For immunostaining, PASMCs were seeded on eight‐well chamber slides and fixed in 4% paraformaldehyde 48 h after transfection. For protein extraction, PASMCs were transfected with GapmeRs or siRNAs, respectively, and whole cell lysates were prepared using 2× Laemmli sample buffer 48 h after transfection. The antibodies used and further details of the procedures are given in the supplementary material, Supplementary materials and methods.

### Subcellular fractionation

RNA for the subcellular fractioning was obtained from PASMCs seeded at a density of 8 × 10^3^ cells/cm^2^ and allowed to grow to 80% confluency in 10‐mm Petri dishes. The precipitation and subsequent purification of the RNA were performed as described elsewhere 33.

### Gene expression analysis

Details of RNA isolation, cDNA synthesis, and quantitative real‐time PCR (qRT‐PCR) analysis are given in the supplementary material, Supplementary materials and methods.

### Fluorescence *in situ* hybridisation

*In situ* hybridisation analysis of *PAXIP1‐AS1* in PASMCs and frozen lung sections (5 μm) was performed using the ViewRNA^®^ Cell Plus Assay (Thermo Fisher Scientific, Waltham, MA, USA). Details may be found in the supplementary material, Supplementary materials and methods.

### Statistics

Mean differences were tested using the two‐sided independent‐sample or paired‐sample *t*‐test. The tests were performed on the logarithms of the concentrations and on the logits of proportions. One‐way analysis of variance (ANOVA) with Tukey's HSD was used to correct for multiple testing. A chi‐square test of independence was performed in case of categorical variables. Values of *p* < 0.05 were considered statistically significant. The *n* number indicates independent experiments. Graphs and statistical calculations were performed using the software package GraphPad Prism Version 5.0 (GraphPad Software, San Diego, CA, USA, RStudio (https://www.rstudio.com) or R (https://www.r-project.org).

Additional information on methods and reagents is available in the supplementary material, Supplementary materials and methods.

## Results

### IPAH small remodelled arteries possess a distinct coding and long non‐coding transcriptional profile

**Figure 1:**
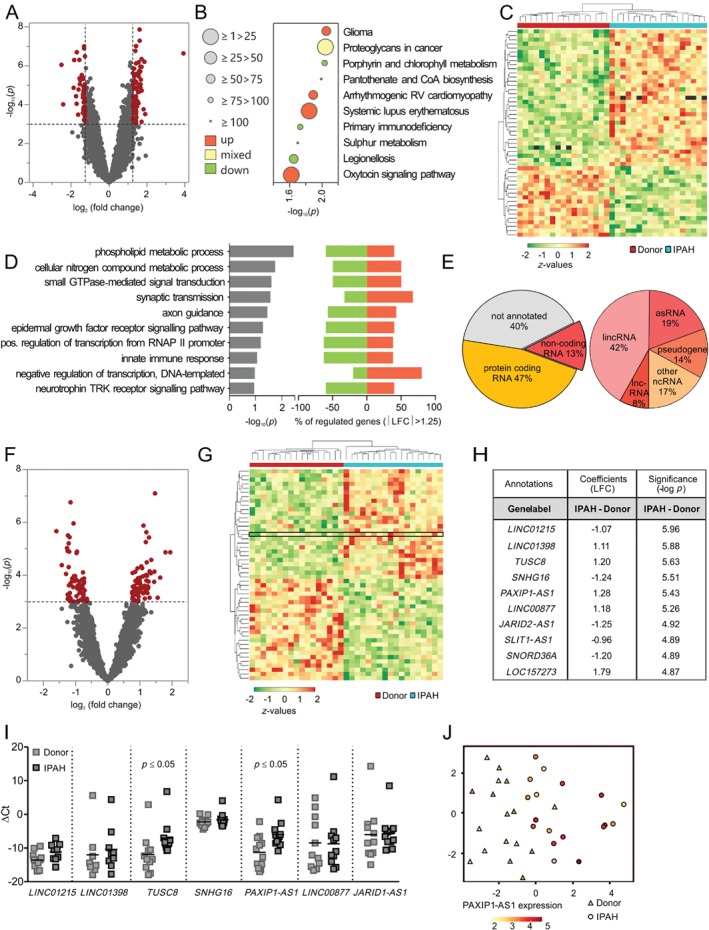
Expression profiling of small pulmonary arteries from IPAH and control donors reveals dysregulation of metabolic, proliferative, and immune‐neuronal pathways and the lncRNA transcriptome. (A) Volcano plot of differentially expressed genes in small pulmonary arteries of IPAH patients. (B) Top ten KEGG pathways after gene set enrichment from all detected genes. (C) Heatmap representing the expression distribution at single patient levels of the 50 most regulated genes. (D) Top ten GO terms (biological processes) after overrepresentation analysis, defined by a minimum significance (–log_10_ *p*) of 3 and a minimum absolute LFC of 1.25. Left, –log_10_ *p* of the perturbation determined from a gene set test; right, percentage of genes from corresponding GO nodes that are up‐ and down‐regulated. (E) Pie‐charts depicting the proportion of protein‐coding and non‐protein‐coding genes, and the categorisation of the non‐coding RNA genes identified in the transcriptome analysis. (F) Volcano plot showing differentially regulated ncRNAs as determined by a minimum –log_10_ *p* of 3. (G) Heatmap representing the expression levels of the 50 most regulated lncRNAs at single patient level. *PAXIP1‐AS1* is highlighted. (H) Annotation and regulation parameters of the top ten regulated lncRNAs in small pulmonary arteries of IPAH patients. (I) qPCR validation of transcriptome analysis. (J) Single patient t‐distributed stochastic neighbour embedding (t‐SNE) analysis showing the distance between single samples with colour‐by‐expression coding of *PAXIP1‐AS1* and respective annotation according to disease.

One hallmark of PAH is the remodelling of small distal pulmonary arteries 34. We defined the gene expression profile in small pulmonary arteries of 18 IPAH patients and 17 controls. Based on a minimum significance (–log_10_ *p*) of 3 and a minimum absolute log_2_ fold change (LFC) of 1.25, 124 annotated protein‐coding genes were classified as up‐ (76) or down‐regulated (48) in IPAH compared with control donor samples (Figure 1A and supplementary material, Table S1). A KEGG pathway analysis following gene set enrichment on the entire dataset indicated a perturbation in metabolic, immunological, neuronal, and proliferative processes in IPAH (Figure 1B and supplementary material, Figure S1A). Connections between regulated genes of the top ten perturbed KEGG pathways are displayed in the supplementary material, Figure S1B. The individual profiles of the 50 genes with the highest significance are shown in the heatmap in Figure 1C. Gene ontology assigned these genes to several nodes, which were overrepresented in IPAH; these predominantly belonged to the groups of phospholipid and nitrogen metabolism, proliferation, immunological and neuronal responses, signal transduction (involving tyrosine receptor kinases and small GTPases), and transcriptional regulation (Figure 1D). In addition to protein‐coding genes, we identified a large number of differentially regulated non‐coding RNAs. This heterogeneous group included long intergenic non‐coding (linc) RNAs, antisense (as) RNAs, pseudogenes, and other long and small ncRNAs, as depicted in Figure 1E. By excluding non‐annotated transcripts and transcripts shorter than 200 nt, we identified a total of 146 regulated non‐coding genes (supplementary material, Table S2). Changes in the expression of non‐coding RNA genes thus contribute substantially to the unique transcriptomic landscape of IPAH vessels (Figure 1F). The lncRNA profiles of IPAH and donor groups were clearly distinct (Figure 1G). The differential expression of seven of the ten most regulated lncRNAs (Figure 1H) was analysed by qRT‐PCR. The lncRNAs *TUSC8* and *PAXIP1‐AS1* were verified to be upregulated in IPAH (Figure 1I). As *PAXIP1‐AS1* was previously reported to be involved in proliferative events 35, we selected it for further analysis. Following a t‐stochastic neighbouring embedding (t‐SNE) dimension reduction of all detected genes, *PAXIP1‐AS1* expression levels at single patient level were visualised in a colour‐by‐expression code. Indeed, the heatmap overlay of *PAXIP1‐AS1* expression resembles the clear discrimination of IPAH and donor transcriptome in the t‐SNE plot (Figure 1J).

### *PAXIP1‐AS1* expression is enhanced in human IPAH‐PASMCs and knockdown reveals its involvement in focal adhesion and ECM–receptor interaction

*PAXIP1‐AS1* comprises a single exon located at chromosome 7q36.2 (position: 155003433–155005703; NC_000007.14, GRCh38.p12 according to the NCBI database). It is flanked on its 5′ side by a GC‐rich region that separates it from its diverging coding gene *PAXIP1* on the opposite strand. To identify whether *PAXIP1‐AS1* expression is limited to the lung, we explored its expression across multiple human tissues using the Genotype‐Tissue Expression (GTEx) portal. Low ubiquitous expression was observed in multiple tissues, with a notable enrichment in the cerebellum (supplementary material, Figure S2A,B). Closer examination of *PAXIP1‐AS1* expression by qRT‐PCR revealed that *PAXIP1‐AS1* was enriched in pulmonary arteries without adventitia, compared with pulmonary veins, bronchi, and pulmonary arterial adventitia (supplementary material, Figure S2C). Furthermore, in isolated cells, *PAXIP1‐AS1* expression was highest in parenchymal fibroblasts and PASMCs, followed by adventitial fibroblasts and pulmonary arterial endothelial cells (supplementary material, Figure S2D).

**Figure 2:**
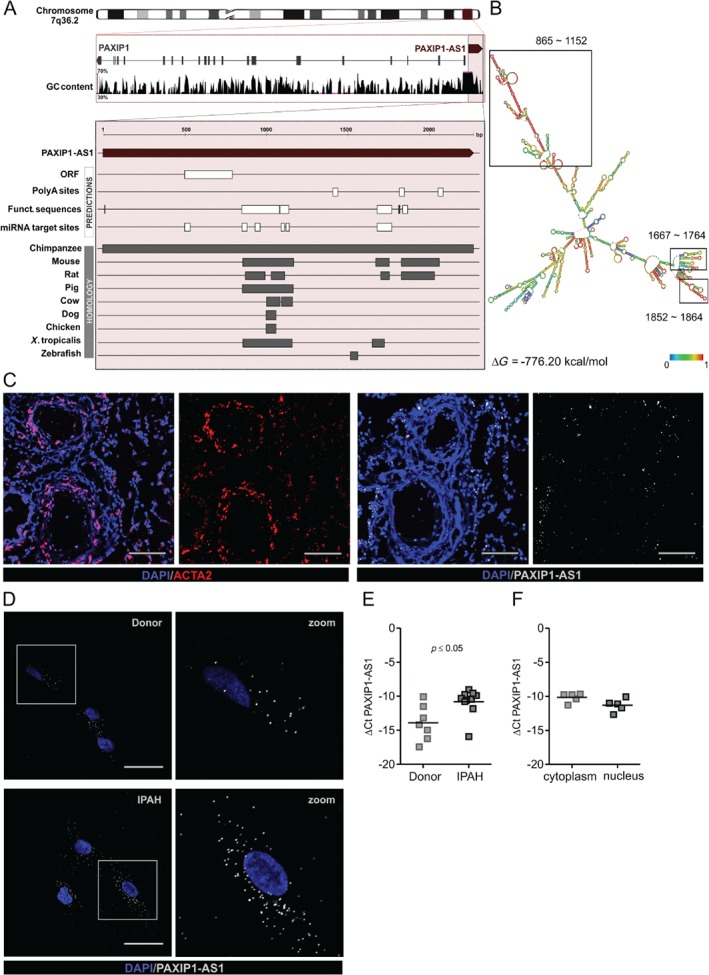
*PAXIP1‐AS1* harbours functional sequences and is abundantly expressed in IPAH‐PASMCs. (A) Chromosomal location and sequence characteristics of *PAXIP1‐AS1* (adapted from entry in UCSC Genome Browser on Human, December 2013 (GRCh38/hg38) Assembly; UCSC ID: uc010lqi.6). Predicted features (white bars) for the transcript of *PAXIP1‐AS1* and conserved regions (grey bars) in exemplary organisms are depicted. Note the occurrence of Alu‐ and AU‐rich elements, and their positional concordance to the conserved regions in various species (see supplementary material, Table S3 for detailed positioning). (B) Predicted secondary structure of lncRNA *PAXIP1‐AS1* calculated using the partition function and base‐pairing probability matrix in addition to the minimum free energy (MFE) structure. The most optimal structure (Δ*G* = −776.20 kcal/mol) is depicted; the approximate Alu‐ and AU‐rich regions are highlighted. Colour intensity denotes base‐pairing probabilities (red = most likely; colour at unpaired regions denotes probability of being unpaired). (C) Representative RNA fluorescence *in situ* hybridisation images of *ACTA1* in red and *PAXIP1‐AS1* in grey on serial cryo sections (5 μm) of IPAH lungs (*n* = 2). Scale bar = 100 μm. (D) Fluorescent images of *PAXIP1‐AS1* (red) RNA *in situ* hybridisation on PASMCs of IPAH and donors (*n* = 2). Scale bar = 50 μm. qRT‐PCR of *PAXIP1‐AS1* in PASMCs of IPAH and donors (E) and after subcellular fractionation of PASMCs (F). *p* ≤ 0.05 as determined by Student's *t*‐test.

Although *PAXIP1‐AS1* harbours a putative open‐reading frame, online coding potential calculators suggested only a weak coding potential (supplementary material, Figure S3A,B and Figure 2A). *PAXIP1‐AS1* structure prediction resulted in a complex alignment and its high stability given by the calculated free energy suggests a functional importance (Figure 2B). We therefore investigated the transcript for functional sequences to further delineate possible biological functions. Several Alu elements, SRP RNA, A‐ and C‐repeats, and an AU‐rich element were identified (Figure 2A and supplementary material, Table S3). Furthermore, the predictions suggest several binding sites for miRNA as well as transcription factors (supplementary material, Tables S4 and S5). Like most lncRNAs 36, *PAXIP1‐AS1* lacks conservation across species beyond primates.

To identify which cells in the pulmonary artery are responsible for increased *PAXIP1‐AS1* levels, we visualised its expression in human IPAH tissue using fluorescence *in situ* hybridisation (FISH). *PAXIP1‐AS1* was expressed in the lung with abundance in α‐smooth muscle actin‐expressing cells (Figure 2C and supplementary material, Figure S4A). The expression of *PAXIP1‐AS1* was further validated in PASMCs isolated from donors and IPAH (Figure 2D,E and supplementary material, Figure S4B). Interestingly, a similar increase was also observed in adventitial, but not parenchymal, IPAH fibroblasts (supplementary material, Figure S2E).

The subcellular localisation of lncRNAs is critical for determining their functional properties. Both FISH and subcellular fractionation experiments detected *PAXIP1‐AS1* in both the nuclear and the cytoplasmic compartments (Figure 2D,F). Next, we checked whether *PAXIP1‐AS1* transcription can be modulated by mediators that are involved in PAH pathogenesis. Treatment of PASMCs with endothelin‐1 (ET‐1) suggested a susceptibility of *PAXIP1‐AS1* expression to the endothelial‐derived mediator (supplementary material, Figure S5).

**Figure 3:**
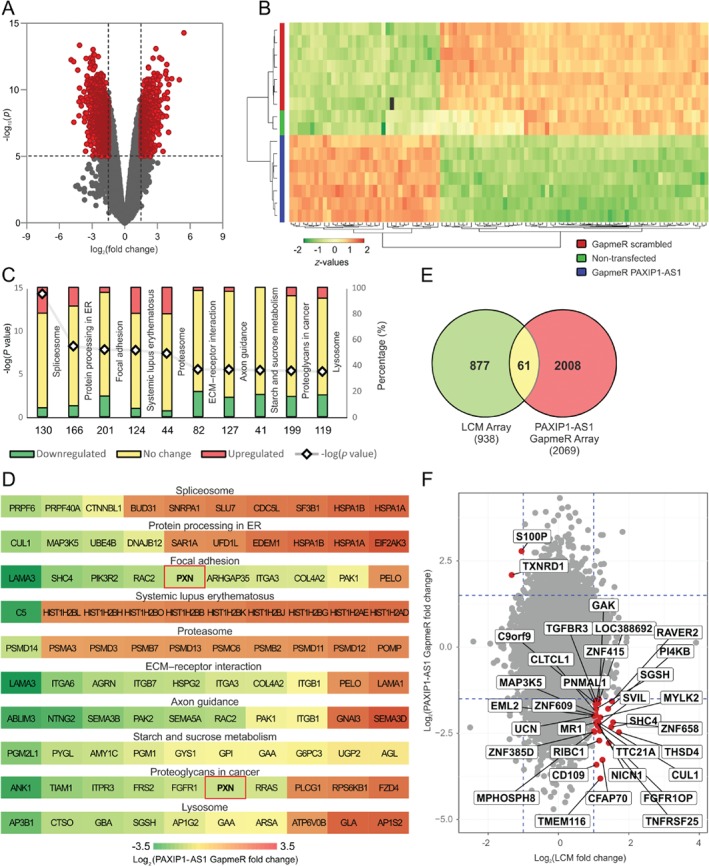
Genes and gene sets dependent on *PAXIP1‐AS1* in IPAH. (A) Volcano plot of log_2_ ratio highlighting differentially expressed genes applying a cut‐off of a minimum –log_10_ *p* of 5 and a minimum absolute LFC of 1.5 after forced knockdown of *PAXIP1‐AS1* on PASMCs. (B) Heatmap representing the top 100 regulated genes. (C) KEGG‐pathway analysis of gene set enrichment of all genes in PASMCs after knockdown of *PAXIP1‐AS1*.–log_10_ *P* values of the perturbation and the percentages of genes from corresponding KEGG pathway that are down‐ and up‐regulated are depicted. (D) Heatmap of the LFC of the ten most regulated genes from the top ten perturbed pathways (as in C). (E) Overlapping regulated genes of transcriptome analysis of arteries obtained by LCM from donor and IPAH and transcriptome analysis after *PAXIP1‐AS1* knockdown in PASMCs. (F) Comparison as in E highlighting inversely regulated genes.

To delineate how *PAXIP1‐AS1* can influence PASMC function, we analysed the gene expression profile following GapmeR‐mediated *PAXIP1‐AS1* knockdown. A clear change in the transcriptional programme was observed, as highlighted by the volcano plot (Figure 3A and supplementary material, Table S6) and in the heatmap representation of the top 100 regulated genes (Figure 3B). Notably, the profiles of untransfected samples were nearly identical to scrambled control‐treated samples (Figure 3B). To gain insights into the global changes induced after *PAXIP1‐AS1* knockdown in PASMCs, we performed a KEGG pathway analysis of all regulated transcripts. *PAXIP1‐AS1* silencing perturbed protein turnover, cytoskeletal arrangement at focal adhesions, extracellular matrix, and pathways implicated in metabolic and proliferative processes (Figure 3C). The genes involved in these pathways are shown in Figure 3D and supplementary material, Table S7. Importantly, *PXN*, encoding the focal adhesion adaptor protein paxillin, was one of the top ten regulated genes in two different KEGG pathways (highlighted in Figure 3D). To delineate how *PAXIP1‐AS1* could exert its effects in IPAH, we investigated potential downstream effectors in IPAH arteries. To this end, we directly compared genes classified as differentially expressed from our two transcriptomic profiling approaches: (1) LCM arteries from IPAH versus donor, and (2) PASMCs after *PAXIP1‐AS1* knockdown. This comparison revealed 61 genes as regulated in common in both approaches (Figure 3E and supplementary material, Table S8). Next, we narrowed the number of genes to 32 by selecting only genes that were regulated in opposite directions, thereby identifying a potential *PAXIP1‐AS1‐*regulated gene set in IPAH. Amongst those, several were implicated in proliferation and apoptosis processes, e.g. the cyclin G‐associated kinase (*GAK*) and cullin 1 (*CUL1*) (Figure 3F).

### *PAXIP1‐AS1* inhibition promoted apoptosis and inhibited PASMC proliferation and migration via its downstream target paxillin

**Figure 4:**
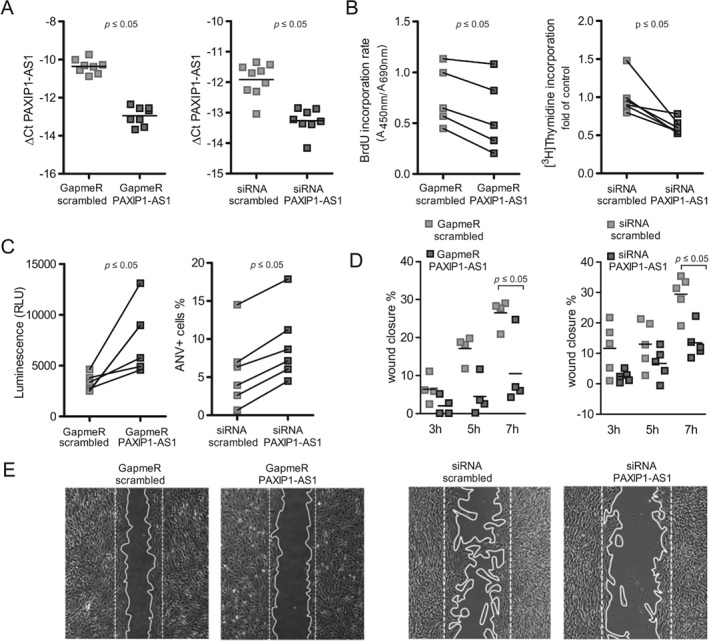
Reduced expression of *PAXIP1‐AS1* interferes with proliferative, apoptotic, and migratory properties of PASMCs. (A) Expression levels of *PAXIP1‐AS1* after LNA‐GapmeR or siRNA‐mediated knockdown as determined by qRT‐PCR. (B) Proliferation of PASMCs determined by BrdU or [^3^H]thymidine incorporation, respectively. (C) Apoptosis measurements in PASMCs determined by luminescence‐based measurement of active caspase 3/7 and flow cytometric AnV/PI staining, respectively. (D) Quantification of a scratch wound‐healing assay to investigate the migratory behaviour of PASMCs at indicated times. (E) Representative pictures (*t* = 7 h of migration) of D. All readouts (A–E) were performed 48 h post‐knockdown. *p* ≤ 0.05 as determined by Student's *t*‐test.

To substantiate the involvement of *PAXIP1‐AS1* in proliferation and apoptosis, we used two complementary loss‐of‐function approaches, GapmeR and siRNA‐mediated knockdown (Figure 4A). Indeed, forced reduction of *PAXIP1‐AS1* potently reduced proliferation and elevated pro‐apoptotic events in PASMCs (Figure 4B,C). As focal adhesions and ECM–receptor interaction pathways were perturbed after *PAXIP1‐AS1* knockdown, we also investigated its role in migratory processes. As observed in a scratch wound healing assay, both knockdown approaches of *PAXIP1‐AS1* resulted in a marked reduction in the migratory potential of PASMCs (Figure 4D,E).

### The *PAXIP1‐AS1*–paxillin axis contributes to the IPAH PASMC phenotype

**Figure 5:**
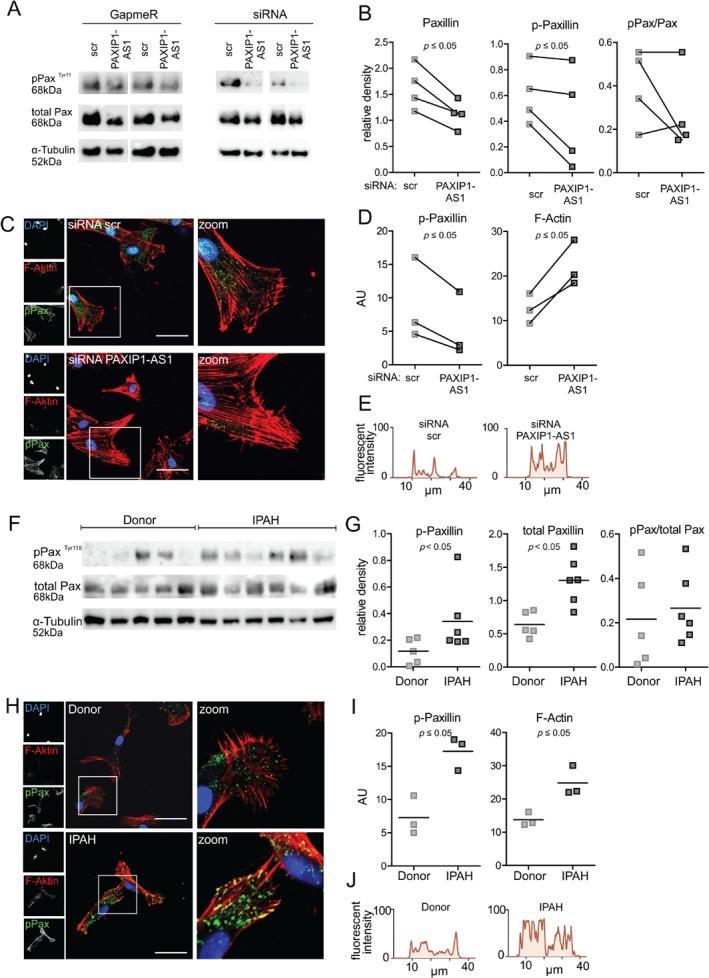
Paxillin is a downstream effector of the lncRNA *PAXIP1‐AS1* in donor PASMCs and is increased in PASMCs from IPAH patients. (A) p‐Paxillin (Tyr118) and total paxillin expression relative to α‐tubulin 48 h after GapmeR‐ or siRNA‐mediated knockdown of *PAXIP1‐AS1* in donor PASMCs, as determined by immunoblotting and quantified by densitometry (B). (C) Immunofluorescence of donor PASMCs 48 h after siRNA‐mediated *PAXIP1‐AS1* knockdown of p‐paxillin (green), F‐actin (phalloidin, red), and nucleus (DAPI, blue). Scale bar = 50 μm. (D) Quantification of fluorescent intensity of p‐paxillin and F‐actin. AU = arbitrary units. (E) Representative F‐actin cross‐sectional fluorescent plots. p‐Paxillin (Tyr118) and total paxillin expression relative to α‐tubulin in PASMCs from IPAH (*n* = 6) and donor (*n* = 5), as determined by immunoblotting (F) and densitometry (G). (H) Immunofluorescence of PASMCs from IPAH and donor of p‐paxillin (green), F‐actin (phalloidin, red), and nucleus (DAPI, blue). Scale bar = 50 μm. All immunofluorescence images are representative of three individual experiments with three individual patients and controls, respectively. *p* ≤ 0.05 as determined by Student's *t*‐test. (I) Quantification of fluorescence intensity of p‐paxillin and F‐actin. AU = arbitrary units. (J) Representative F‐actin cross‐sectional fluorescence plots. *p* ≤ 0.05 as determined by Student's *t*‐test.

Changes in focal adhesions can contribute to disease progression. To delineate the intrinsic mechanism of *PAXIP1‐AS1* on changes in focal adhesions, we focused on paxillin as a downstream effector of *PAXIP1‐AS1* in healthy donor PASMCs. We confirmed that depletion of *PAXIP1‐AS1* resulted in decreased total and phospho (p)‐paxillin protein levels (Figure 5A,B). Since precise coordination between focal adhesions and the actin cytoskeleton is essential for cell migration, we visualised p‐paxillin together with F‐actin on donor PASMCs. After *PAXIP1‐AS1* knockdown, p‐paxillin levels were decreased, whereas F‐actin levels increased (Figure 5C,D). Plotting of the cross‐sectional F‐actin signal suggested cytoskeletal rearrangement and increased stress fibre formation visualised in the number and width of peaks in response to the knockdown of *PAXIP1‐AS1* (Figure 5E and supplementary material, Figure S6A,B). This suggests that knockdown of *PAXIP1‐AS1* and the reduced levels of paxillin trigger a stress response in the cells. Interestingly, the focal adhesion kinase (FAK), another focal adhesion protein, showed a similar tendency to downregulation after knockdown of *PAXIP1‐AS1* (supplementary material, Figure S7A–C). Next, we translated these findings into the patient context, and investigated paxillin in IPAH PASMCs. We detected increased total and p‐paxillin protein levels, but no robust changes in mRNA expression in IPAH PASMCs compared with donors (Figure 5F,G and supplementary material, Figure S8A). In IPAH PASMCs, the increased levels of p‐paxillin were accompanied by an increase in F‐actin levels and appeared locally associated with both ventral and distal stress fibres (Figure 5H–J, and supplementary material, Figure S6C,D). Collectively, this suggested a link between the lncRNA *PAXIP1‐AS1* and IPAH PASMC function that depends on the cytoskeleton and the focal adhesion protein paxillin.

**Figure 6:**
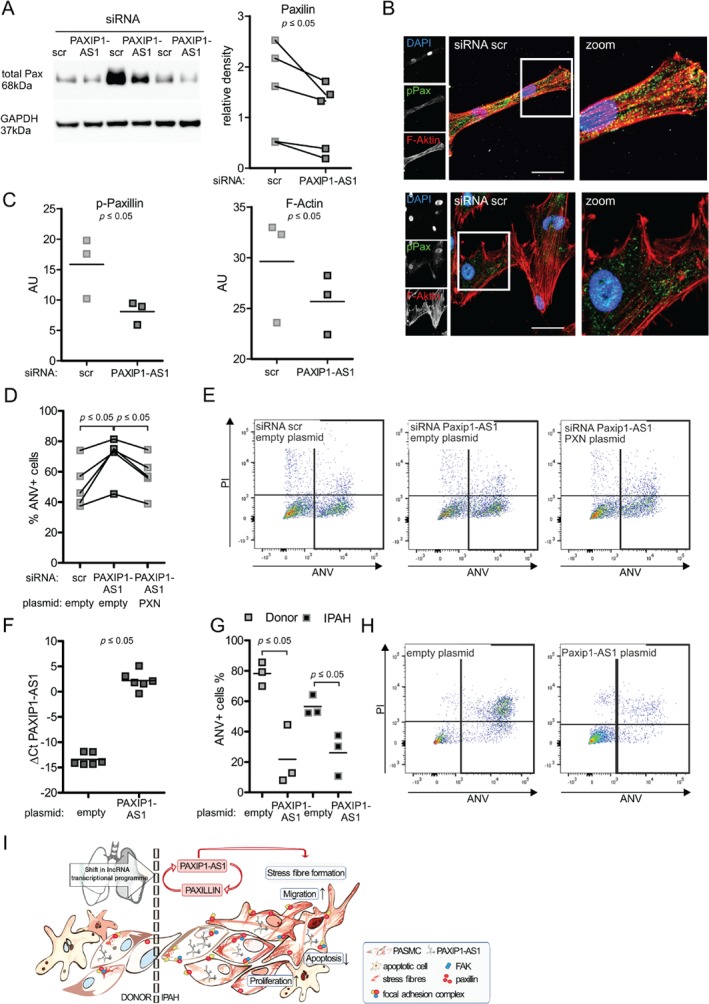
*PAXIP1‐AS1* mediates its effect on apoptosis via paxillin in IPAH PASMCs. (A) Total paxillin levels relative to GAPDH 48 h after siRNA‐mediated knockdown of *PAXIP1‐AS1* in IPAH PASMCs, as determined by immunoblotting and densitometry. *n* = 5. (B) Immunofluorescence of IPAH PASMCs 48 h after siRNA‐mediated *PAXIP1‐AS1* knockdown of p‐paxillin (green), F‐actin (phalloidin, red), and nucleus (DAPI, blue). Scale bar = 50 μm. (C) Quantification of fluorescence intensity of p‐paxillin and F‐actin. AU = arbitrary units. (D) Apoptosis measurements in IPAH PASMCs determined by flow cytometric AnV/PI staining 48 h after siRNA‐mediated *PAXIP1‐AS1* knockdown and co‐transfection with empty or *PXN* overexpression plasmid. (E) Representative flow cytometric scatter plots. (F) Expression levels of *PAXIP1‐AS1* 48 h after transfection with *PAXIP1‐AS1* overexpression plasmid in donor and IPAH PASMCs. (G) Apoptosis measurements in donor and IPAH PASMCs determined by flow cytometric AnV/PI staining 48 h after transfection with *PAXIP1‐AS1* overexpression plasmid. (H) Representative flow cytometric scatter plots. (I) Schematic model of *PAXIP1‐AS1* regulation on PASMC function in IPAH. *p* ≤ 0.05 as determined by Student's *t*‐test, one‐way ANOVA followed by Dunnett's multiple comparison test, or two‐way ANOVA followed by Bonferroni post‐test.

To further examine this link, we next performed *PAXIP1‐AS1* knockdown experiments in IPAH PASMCs. Here, *PAXIP1‐AS1* knockdown robustly reduced total paxillin protein levels, while F‐actin levels were unaffected (Figure 6A–C). Knockdown of *PAXIP1‐AS1* in IPAH PASMCs led to increased apoptotic susceptibility, which was rescued by simultaneous overexpression of paxillin (Figure 6D,E and supplementary material, Figure S8B,C). While knockdown of *PAXIP1‐AS1* robustly decreased total protein paxillin levels, we could not observe consistent changes in the gene expression levels of paxillin (supplementary material, Figure S8C). Next, we overexpressed *PAXIP1‐AS1* in both donor and IPAH PASMCs (Figure 6F) and investigated its effect on apoptosis resistance. Overexpression of the lncRNA *PAXIP1‐AS1* potently decreased the apoptosis susceptibility in both donor and IPAH PASMCs (Figure 6G,H), suggesting a role in IPAH‐related cellular dysfunction (Figure 6I).

## Discussion

To date, transcriptomic profiling of IPAH patients either has often lacked compartment‐specific analysis or has been limited by the number of patients analysed. Here, we not only describe an IPAH‐specific transcriptome in small remodelled arteries in a comparable large cohort of patients, but also address the potential link between dysfunctional lncRNA regulatory mechanisms and the transcriptional changes implicated in vascular remodelling.

In our compartment‐specific gene expression analysis, we identified key transcriptional pathways that were perturbed in IPAH; those included metabolic, neuronal, proliferative, and immunological processes. These processes are tightly interconnected. In IPAH, increased proliferation and migration of SMCs can be associated with metabolic changes 37, 38. Adaptions of metabolic processes might be necessary to keep up with the increased energy expenditure or, vice versa, increased energy utilisation might induce a hyperproliferative phenotype. Interestingly, neuronal 39, 40 as well as inflammatory mediators 41 can influence a cell's metabolism and growth response, and immunological as well neuronal components have been described in IPAH 42, 43. Given this tight connection of deregulated pathways in remodelled arteries in IPAH, it is apparent that targeting one mechanism might not be sufficient to induce reverse vascular remodelling. In contrast, upstream control mechanisms which simultaneously affect multiple transcriptional pathways might be able to overcome this limitation.

LncRNAs have emerged as critical determinants in human diseases. They can regulate gene expression at epigenetic, transcriptional, and translational levels. Thus, changes in the lncRNA expression profile can be associated with pathological alterations within the coding transcriptome. In this respect, lncRNAs have great capacity for gene regulation 44. In pulmonary vascular remodelling, little is known about the involvement of lncRNAs in disease development and progression. Only a few recent studies have shown their contribution to vascular smooth muscle cell proliferation or apoptosis (e.g. *MALAT1* 45 or *LnRPT* 46) and only one study has investigated the lncRNA profile in PAH patients using peripheral blood lymphocytes 47. Here, we have demonstrated that transcriptional reprogramming in small remodelled arteries (intima and media) is associated with a specific lncRNA expression profile in IPAH. Importantly, the lncRNA transcriptome can be highly cell type and disease‐specific 48, and thus can be used to stratify disease subtypes or monitor disease progression.

Among many differentially regulated lncRNAs, we identified *PAXIP1‐AS1* as strongly regulated in IPAH small pulmonary arteries, PASMCs, and adventitial fibroblasts. In general, *PAXIP1‐AS1* expression was not limited to the lung or any specific cell type, which differs from other reported ncRNAs, e.g. miR206 or miR126 49, 50, 51. Under physiological conditions, *PAXIP1‐AS1* expression is ubiquitous but comparably very low. Its upregulation during disease therefore suggests a crucial role. In that regard, we identified *PAXIP1‐AS1* as a critical regulator of PASMC function. The function of a given lncRNA depends on its subcellular localisation and the presence of functional RNA sequences on the transcript. In PASMCs, *PAXIP1‐AS1* was ubiquitously localised in the nucleus and in the cytoplasm, and its secondary structure harboured several functional elements. *PAXIP1‐AS1* contained several Alu elements in two separate regions. Alu elements belong to the group of short interspersed repeated sequences (SINEs) and are mostly associated with function in the cytoplasm, where they mediate mRNA stability 52. The *PAXIP1‐AS1* transcript also harboured miRNA binding sites. Sponging of miRNAs is another feature that is associated with a cytoplasmic function of lncRNAs. We also identified A‐ and C‐rich tracts on the *PAXIP1‐AS1* transcript. These tracts can potentially infer transcriptional regulation, either via epigenetic modulation or via interaction with transcription factors (TFs) 53, 54. Indeed, several binding sites for TFs were revealed on the *PAXIP1‐AS1* transcript. The list included some implicated in PH, such as HIF2α, HIF1α, NFAT, NFκB, and FOXO1 55. In general, the interaction of an lncRNA with a TF can be multifunctional; it can either inhibit gene expression by acting as a decoy for TF binding or facilitate gene expression by promoting TF accessibility/recruitment to enhancer elements 56. Taken together, it is apparent that certain structural RNA elements can exert different functional effects depending on the subcellular localisation of the lncRNA. This spatio‐temporal action of lncRNAs also applies to *PAXIP1‐AS1*. In comparison to PASMCs, where *PAXIP1‐AS1* is located both in the cytoplasm and in the nucleus, it was found to be retained in the nucleus in HEK‐293 cells 35. This resulted in functional characteristics differing from our observation, such as opposite proliferative effects.

In PASMCs, *PAXIP1‐AS1* knockdown interfered with the PAH‐related cellular function by inhibiting migration and proliferation, while increasing apoptotic susceptibility. Accordingly, overexpression of *PAXIP1‐AS1* in both donor and IPAH PASMCs led to apoptosis resistance. This suggests that *PAXIP1‐AS1* can potentially modulate PASMC‐mediated vascular remodelling events. The key pathways that were dependent on *PAXIP1‐AS1* and strongly perturbed after knockdown were ECM–receptor interaction and focal adhesions. Both pathways are tightly interconnected, as cell motility depends on a functional interaction of focal adhesions with the surrounding extracellular matrix. Thus, changes in focal adhesions are central to the migratory and proliferative maladaptation of PASMCs in PAH. Indeed, paxillin and other adapter proteins of the focal adhesion complex are strongly associated with IPAH pathology 57, 58, 59. Our transcriptomic analysis and complementary cellular assays identified paxillin as a downstream target of *PAXIP1‐AS1*. Knockdown of *PAXIP1‐AS1* potently reduced paxillin levels and concomitantly induced a cytoskeletal disruption in donor and IPAH PASMCs, which was accompanied by increased apoptotic susceptibility. Overexpression of *PXN* reversed this effect, and simultaneously increased *PAXIP1‐AS1* expression levels (supplementary material, Figure S8A). This suggests a reciprocal dependency of the PAXIP1‐AS1–paxillin axis in the mediation of the IPAH phenotype. Interestingly, in addition to paxillin, *PAXIP1‐AS1* knockdown also resulted in decreased FAK levels. In IPAH, it has previously been observed that FAK and paxillin levels are co‐regulated 59 – a notion substantiated by this study. Together, this suggests that *PAXIP1‐AS1* knockdown induced an impairment of the focal adhesion axis, from integrins to actin filaments. While targeting cell motility in vascular remodelling seems promising at first, a loss of function of any member of the focal adhesion complex might be deleterious, due to their involvement in fundamental cellular processes.

In addition to focal adhesion, lncRNA *PAXIP1‐AS1* also controlled other critical pathways of PAH pathology. Among the most significantly downregulated pathways after *PAXIP1‐AS1* knockdown in PASMCs were also proliferative processes, axon guidance, and sugar metabolism. Interestingly, these pathways mirror the processes that we identified in the compartment‐specific transcriptomic analysis of small remodelled arteries. These findings indicate that (1) these processes are pivotal to the IPAH pathology, (2) IPAH‐specific pathological changes are strongly associated with a tightly regulated transcriptional programme, and (3) lncRNAs can act as important regulators in these cellular events in IPAH pathology.

At the moment, it is still unclear whether *PAXIP1‐AS1* is causative in disease development or merely a consequence of remodelling processes. Two major limitations of this study in answering this question are (1) its lack of sequence conservation in animals, and (2) the use of human transplanted lungs that always reflect end‐stage disease.

Taken together, a multitude of pathways and single molecules finally converge in the manifestation of the maladaptive cellular phenotype in IPAH. The identification of lncRNA *PAXIP1‐AS1* as a critical component of the dysfunctional control machinery adds another piece to the complex patho‐mechanism of IPAH.

## Author contributions statement

KJ, HTP, LMM, MB, and GK conceived the study and drafted the manuscript. KJ, HTP, LMM, JW, and JH carried out experiments and performed data analysis. KS and WK performed data collection. HO and AO contributed to study design and data interpretation.

**SUPPLEMENTARY MATERIAL ONLINE**

**Supplementary materials and methods**

**Supplementary figure legends**

**Figure S1**. Enriched KEGG pathways in LCM array

**Figure S2**. *PAXIP1‐AS1* expression in various tissues

**Figure S3**. Coding potential assessment

**Figure S4**. *PAXIP1‐AS1 in situ* hybridisation

**Figure S5**. *PAXIP1‐AS1* after cytokine stimulation

**Figure S6**. Cross‐sectional F‐actin plots

**Figure S7**. *PAXIP1‐AS1* influences FAK expression

**Figure S8**. *PAXIP1‐AS1* and PXN expression

**Table S1.** Differentially regulated coding genes in IPAH

**Table S2.** Differentially regulated non‐coding genes in IPAH

**Table S3.** Functional regions in *PAXIP1‐AS1*

**Table S4.** Transcription factor binding sites in the *PAXIP1‐AS1* transcript

**Table S5.** miRNA target sites in the *PAXIP1‐AS1* transcript

**Table S6**. *PAXIP1‐AS1* knockdown: differentially regulated genes

**Table S7**. *PAXIP1‐AS1* knockdown: list of genes from the top regulated pathways

**Table S8.** Comparative transcriptomic analysis: differentially regulated genes

**Table S9.** Primer sequences (mentioned in the supplementary material, Supplementary materials and methods)

## Supporting information


**Supplementary materials and methods**

**Supplementary figure legends**

**Figure S1**. Enriched KEGG pathways in LCM array. (A) Summary of barcode plots, showing the individual statistics of the most significantly regulated KEGG pathways. (B) Representation of most significant genes from the regulated KEGG pathways and representation of a minimum network analysis as performed by NetworkAnalyst (protein–protein interaction by STRING interactome with confidence score cut‐off of 900) connecting the pathways.

**Figure S2**. *PAXIP1‐AS1* expression in various tissues. The expression landscape of *PAXIP1‐AS1* depicted here (A, general overview; B, study‐relevant selection) is obtained from the GTEx Portal on 1 August 2018 and has the dbGaP accession number phs000424.v7.p2. (C) PAXIP1‐AS1 expression in selected study‐relevant tissues (*n* = 1), (D) isolated cells and (E) in donor and IPAH adventitial and parenchymal fibroblasts. *P* ≤ 0.05 as per Student's *t*‐test.

**Figure S3**. Coding potential assessment. The coding ability of the *PAXIP1‐AS1* transcript was calculated by available online coding potential assessment tools (A) CPC (Coding Potential Calculator) and (B) CPAT (Coding Potential Assessment Tool).

**Figure S4**. *PAXIP1‐AS1* in situ hybridization. The fluorescent in situ hybridization images show *PAXIP1‐AS1*‐stained lung tissue (A) and PASMC (B) together with the appropriate control stainings. Scale bar = 100 μm for (A) and 50 μm for (B).

**Figure S5**. *PAXIP1‐AS1* after cytokine stimulation. qRT‐PCR of *PAXIP1‐AS1* in PASMC after cytokine stimulation of PASMC for indicated times. *P* ≤ 0.05 as per one‐way ANOVA and Dunnett's post hoc test.

**Figure S6**. Cross‐sectional F‐actin plots. Display of single cell cross‐sectional analysis of F‐actin fluorescence intensity signal, in donor PASMC 48 h following transfection with (A) siRNA scrambled or (B) siRNA *PAXIP1‐AS1*. Single cell cross‐sectional analysis of F‐actin fluorescent intensity signal in (C) donor PASMC and (D) IPAH PASMC.

**Figure S7**. *PAXIP1‐AS1* influences *FAK* expression. (A) Immunofluorescence of IPAH PASMC 48 h after siRNA‐mediated *PAXIP1‐AS1* knockdown; FAK (green), F‐actin (phalloidin, red) and nucleus (DAPI, blue); scale bar = 50 μm. (B) Quantification of fluorescence intensity of FAK; AU = arbitrary units. (C) FAK levels relative to GAPDH 48 h after siRNA‐mediated knockdown of *PAXIP1‐AS1* in IPAH PASMC, as determined by immunoblotting and densitometry, *n* = 5 (same samples but different blot as used in Figure 6A). *P* ≤ 0.05 as determined by Student's *t*‐test.

**Figure S8**. *PAXIP1‐AS1* and *PXN* expression. (A) *PXN* gene expression in isolated PASMC from donor and IPAH patients, determined by qRT‐PCR. (B) *PAXIP1‐AS1* and (C) *PXN* gene expression levels, determined by qRT‐PCR 48 h after siRNA‐mediated *PAXIP1‐AS1* knockdown and co‐transfection with empty or *PXN* overexpression plasmid.

**Table S1**. Differentially regulated coding genes in IPAH

**Table S2**. Differentially regulated non‐coding genes in IPAH

**Table S3**. Functional regions in *PAXIP1‐AS1*

**Table S4**. Transcription factor binding sites in the *PAXIP1‐AS1* transcript

**Table S5**. miRNA target sites in the *PAXIP1‐AS1* transcript

**Table S6**. *PAXIP1‐AS1* knock‐down: differentially regulated genes

**Table S7**. *PAXIP1‐AS1* knock‐down: list of genes from top regulated pathways

**Table S8**. Comparative transcriptomic analysis: differentially regulated genes

**Table S9**. Primer sequences
