# Supplementary material for: Long non‐coding RNAs influence the transcriptome in pulmonary arterial hypertension: the role of PAXIP1‐AS1
Source: J Pathol. 2019 Jan 16;247(3):357–70. doi: 10.1002/path.5195 (PMC6900182; doi:10.1002/path.5195)
Supplement: Supplementary file 1 — Supplementary materials and methods [file PATH-247-357-s001.docx]

**Supplementary materials and methods**

**Patient samples**

Human lung tissue samples were obtained from IPAH patients who underwent lung transplantation at the Department of Surgery, Division of Thoracic Surgery, Medical University of Vienna, Austria. The institutional ethics committee approved the protocol and tissue usage (976/2010), and patient consent was obtained before lung transplantation. Downsized non-tumourous, non-transplanted donor lungs served as controls. Information on the patients’ key clinical characteristics can be found in Table 1. A detailed description on how the explanted lungs were sampled and stored can be found in ref 60.

**Genome-wide expression profiling**

Whole genome expression profiling was performed on material obtained from laser capture micro-dissected small pulmonary arteries of IPAH patients.

Total RNA extracts were purified from the LCM pulmonary arteries ranging from 50 to 500 µm in diameter (intima and media) of IPAH patients (*n* = 18) and controls (*n* = 17). The RNA was amplified using the Ovation PicoSL WTA System V2 kit (NuGEN Technologies, Bemmel, The Netherlands) before proceeding with labelling using 2 µg of the amplified product. For delineating PAXIP1-AS1 downstream signalling, total RNA extracts were gathered from untreated (GapmeR_nontransfected; *n* = 2), non-targeting GapmeR-treated (GapmeR_negative; *n* = 7), and PAXIP-AS1 GapmeR-treated (GapmeR_PAXIP1AS1; *n* = 7) samples. Further, 150–200 ng of RNA per sample was taken for labelling.

The SureTag DNA labelling kit (Agilent, Waldbronn, Germany) was used to Cy5- and Cy3-label the samples which were subsequently hybridised to 8 × 60K 60mer oligonucleotide spotted microarray slides (SurePrint G3 Human GE v3 8 × 60K Microarray; Agilent Technologies, design IDs: 072363 and 039494). The following hybridisation, washing, and drying steps were performed following the Agilent hybridisation protocol. Thereafter, the slides were scanned at 2 µm/pixel resolution using the InnoScan 900 (Innopsys, Carbonne, France). Image analysis was performed with GenePix Pro 5.1 software, and the calculated values for all spots were saved as GenePix result files. The data were analysed using R (3.3.2) and the limma package (3.30.13) from BioConductor [[61,62](#_ENREF_2)]. Background correction was conducted using the NormExp procedure on the negative control spots and the data were quantile-normalised before averaging [[63](#_ENREF_4)]. Binary logarithms of the mean spot signals were taken for further analysis. The ranking of the genes was performed based on a moderated t-statistics, and pathway analyses were performed using gene sets on the ranks of the t-values.

For the LCM transcriptomic analysis, all protein coding genes with a log_2_ fold change [LFC; defined as the ratio between the signal intensities (B/A)] > 1.25 and –log_10_ *p* > 3 were considered as differentially expressed. For the non-coding genes, a threshold of LFC > 1 and – log_10_ *p* > 3 was chosen. In the GapmeR PAXIP1-AS1 knockdown transcriptomic analysis, all genes with a threshold of LFC > 1.5 and – log_10_ *p* > 5 were considered as differentially expressed. More details may be found in the study by Hoffmann *et al* [[63](#_ENREF_1)]. Log_2_ LFCs in IPAH versus donor are expression changes relative to the donor, and LFCs in the KD array are changes in expression after PAXIP1-AS1 GapmeR silencing relative to the non-targeting GapmeR-treated control. The Rtsne package (0.13) implemented in R (with perplexity parameter set to 7) was used to perform dimensional reductions based on t-SNE.

**Bioinformatic analysis**

Potential functional RNA motifs and binding sites in PAXIP1-AS1 were studied using an integrated web server, namely RegRNA 2.0 at http://regrna2.mbc.nctu.edu.tw/ [64]. The web-based interfaces of CPC at http://cpc.cbi.pku.edu.cn and CPAT at http://lilab.research.bcm.edu/cpat/ were used to calculate the coding potentials of the lncRNAs [[6](#_ENREF_6)5,66]. The Vienna RNA Websuite was used for nucleic acid folding and thermodynamic ensemble prediction of PAXIP1-AS1 [6[7](#_ENREF_8)]. For sequence similarity search, we ran BLAST, version 2.7.1 [[68,69](#_ENREF_9)] and relied on the NCBI nonredundant database [70].

The NetworkAnalyst and Cytoscape 3.6.1 programs for network-based visual analytics were used to explore the interactions between core interacting proteins of the regulated KEGG pathways [71].

**Cell culture**

Primary human PASMCs were either bought from ScienCell (ScienCell Research Laboratories, Carlsbad, CA, USA) or isolated from pulmonary arteries from non-transplanted donor lungs or IPAH lungs (purity ≥ 95% smooth muscle-specific isoforms of α-actin-positive cells). The cells were incubated in a humidified atmosphere at 37°C and 5% CO_2_ and cultured in VascuLife^®^ SMC Medium (LifeLine Cell Technology, Frederick, MD, USA) containing 5% FBS, 5 ng/ml FGF, 5 µg/ml insulin, 50 µg/ml ascorbic acid, 10 mm l-glutamine, 5 ng/ml EGF, 30 mg/ml gentamicin, and 15 µg/ml amphotericin B. Cells with passage numbers between 2 and 8 were taken for the experiments.

**Transient transfection of primary cells**

For manipulation of endogenous levels of PAXIP1-AS1, PASMCs were either transfected with GapmeR antisense oligonucleotides (custom LNA oligonucleotide, Design ID: 657824; Exiqon, Vedbaek, Denmark; 25 nm) or siRNAs (Lincode Human PAXIP1-AS1 siRNA, N-184835-05-0005; Dharmacon, Vienna, Austria; 50 nm) using the transfection reagents Lipofectamine 2000 (Thermo Fisher Scientific, Waltham, MA, USA) or jetPrime (Polyplus Transfection, Illkirch-Graffenstaden, France), respectively. Cells transfected with a non-gene-targeting GapmeR (custom LNA oligonucleotide, Design ID: 657823; Exiqon; 25 nm) or siRNA (ON-TARGETplus non-targeting siRNA #1, D-001810-01-05; Dharmacon; 50 nm) sequences served as controls.

For construction of the PAXIP1-AS1 overexpression plasmid, we amplified full-length PAXIP1-AS1 by PCR using the primers F: 5′-GCTGGCTAGCGCGCGCGGCGGAGGG-3′ and R: 5′-GCTGAAGCTTTAAAAAGGCAGCATGATCTGTATTTGG-3′, and cloned into pcDNA3.1 between NheI and HindIII. Paxillin overexpression plasmid was used in a previous study [72]. Plasmid transfection in PASMCs was performed using the jetPrime (Polyplus Transfection) transfection reagent. An empty pcDNA3.1 vector served as a control, and pmaxGFP™ Vector (Lonza) was taken to co-transfect in order to be able to select for positively transfected cells.

**Immunofluorescence staining**

For immunostaining, PASMCs were seeded on eight-well chamber slides to a density of 8000 cells per well and fixed in 4% paraformaldehyde 48 h post-transfection. After blocking with 5% BSA (Sigma-Aldrich, Vienna, Austria) in PBS, cells were incubated overnight at 4°C with anti-pPaxillin (Tyr118, #2541; Cell Signaling; 1:50) and anti-FAK (3285, Cell Signaling; 1:200). Donkey anti-rabbit 488, 1:500 (for pPaxillin and FAK) were used as secondary antibodies. F-actin staining was performed with the labelled antibody Alexa Fluor 555-Phalloidin (A34055; Thermo Fisher Scientific; 1:80). Finally, the slides were preserved using a mounting medium containing 4′,6-diamidino-2-phenylindole dihydrochloride (DAPI) (Vectashield, Vector Laboratories). Fluorescent intensity was quantified as intensity/area. F-actin intensity profiles were obtained by placing a line of 50 µm along the cross-section of pulmonary arterial smooth muscle cells and plotting the intensity profiles. Sharp distinct peaks represent individual stress fibres and the width of the peak indicates the width of a stress fibre [73]. At least five individual cells from each condition *n* = 3 were analysed. Quantification of immunofluorescent intensity and profiles were performed in ImageJ 1.46r.

**Protein isolation and western blot analysis**

For protein extraction, PASMCs were transfected with GapmeRs or siRNAs, respectively, and whole cell lysates were prepared using 2× Laemmli sample buffer. The protein samples were run in a 10% SDS polyacrylamide gel and transferred to a 0.45-µm PVDF membrane (Immobilon-P, Millipore Corporation). Primary antibodies used for immunoblotting were incubated overnight at 4°C (anti-pPaxillin Tyr118, 2541, Cell Signaling; 1:1000; anti-FAK, 3215, Cell Signaling, 1:1000; anti-α-tubulin, 2125S, Cell Signaling, 1:5000; and anti-GAPDH, CAB932Hu22, Cloud Clone Corp, 1:2000). Detection was performed using the Amersham ECL Plus Western Blotting Detection System (GE Healthcare). ImageJ 1.46r was used for the densitometric analysis of the blots.

**Cell stimulation**

In order to investigate the response to different stimuli, PASMCs were grown on 12-well plates and starved overnight in VascuLife Basal Medium (0% FCS and 0.2% antibiotic/antimycotic). Subsequently, the cells were treated with PDGF-BB (Sigma-Aldrich; 10 ng/ml), TNF-α (eBioscience, Wien, Austria; 10 ng/ml), IL-1β (PeproTech, Wien, Austria; 10 ng/ml), TGF-β (ImmunoTools, Friesoythe, Germany; 10 ng/ml) or endothelin-1 (Sigma-Aldrich; 500 nm), respectively, and total RNA was collected 2, 4, 8, and 24 h post-treatment. The change in expression was measured via qRT-PCR and evaluated relative to an untreated control sample.

**Subcellular fractionation**

To distinguish the presence of lncRNAs in the nucleus and cytoplasm, subcellular fractioning of PASMCs was performed as described elsewhere [74]. PASMCs were seeded in 100-mm Petri dishes at a density of 8 × 10^3^ cells/cm^2^. Precipitated RNA of the nucleus and cytoplasm were lysed in 700 µl of Qiazol (Qiagen, Hilden, Germany). Subsequent purification of the obtained fractionated RNA was carried out using the miRNeasy Mini Kit (Qiagen).

**RNA isolation and cDNA synthesis**

Total RNA of cultured cells was purified using the RNeasy MiniPrep Kit (Qiagen) or peqGOLD total RNA isolation kit (PeqLab, Erlangen, Germany). The quality of isolated RNA was assessed by spectrophotometric analysis (Nanodrop). Total RNA was reverse-transcribed using GoScript™ Reverse Transcriptase Mix with random hexamers (Promega, Dübendorf, Switzerland) or the iScript cDNA Synthesis Kit (BioRad, Vienna, Austria).

**Quantitative real-time PCR (qRT-PCR) analysis**

SYBR Green qRT-PCR was carried out to quantify specific gene transcripts using the StepOnePlus system (Applied Biosystems, Thermo Fisher Scientific) or the CFX384 Touch™ Real-Time PCR Detection System (Biorad). The sequences of the primers used for gene expression analysis are shown in the supplementary material, Table S9. Specific amplification was verified by melt curve analysis. The expression of β-actin (ACTB) or β2-microglobulin (B2M) was used to normalise the obtained expression levels of genes of interest. Differential gene expression was calculated using the threshold cycle (Ct) method [75]. The primer sequences are listed in the supplementary material, Table S9.

**Fluorescence *in situ* hybridisation**

The ViewRNA^®^ Cell Plus Assay (Thermo Fisher Scientific) was used for *in situ* hybridisation analysis of PAXIP1-AS1 in PASMCs and frozen lung sections (5 µm). For PASMCs, the assay was performed according to the manufacturer’s instructions in the ViewRNA^®^ Cell Plus Assay protocol. For tissues, the ViewRNA™ ISH Tissue 1-Plex Assay protocol by Thermo Fisher Scientific was followed until the digestion step. For digestion, the tissue sections were successively incubated for 10 min at room temperature in proteinase K (1 mg/ml in PBS; 04-1070, Peqlab) and in 0.3% Triton X-100 (T8787, Sigma-Aldrich). Target probe hybridisation and signal amplification steps were then performed according to the ViewRNA^®^ Cell Plus Assay protocol. The probe sets for PAXIP1-AS1 (VA1-3015586, Type I, Thermo Fisher Scientific) and ACTA2 (VA6-13283, Type 6, Thermo Fisher Scientific) were used at a dilution of 1:10. Cells were mounted using the Dako Fluorescence Mounting Medium (Agilent Technologies). The microscopic detection was performed on Nikon’s A1+ confocal laser microscope system.

**Migration assay**

Migration of PASMCs was employed in a modified *in vitro* wound-healing assay. Briefly, 15 000 cells were seeded on each side of an Ibidi culture insert μ-dish (Ibidi, Munich, Germany) and transfected as described above. The inserts were removed 48 h post-transfection to create a gap of approximately 500 μm in width. The closing gaps were photographed at 4× magnification (Olympus CKX41) at the indicated time points and the migration rate of PASMCs was quantitatively assessed comparing the initial gap with the area of the healing wound using image analysing software (ImageJ 1.46r).

**Proliferation assay**

The PASMC proliferation rate was determined either by 5-bromo-2-deoxyuridine (BrdU) (Roche Diagnostics, Mannheim, Germany) or by [^3^H]thymidine (BIOTREND Chemikalien GmbH, Cologne, Germany) incorporation assays. PASMCs were seeded in 96-well plates at a density of 5000 cells per well, and BrdU or [^3^H]thymidine, respectively, was added to each well 24 h post-transfection. After an additional incubation period of 24 h, the incorporated BrdU was detected using the colorimetric BrdU assay by Roche and [^3^H]thymidine incorporation was measured as radioactivity by a scintillation counter (Wallac 1450 MicroBetaTriLux Liquid Scintillation Counter and Luminometer) and used as indices of DNA synthesis. The experiments were in both cases performed in quadruplicates.

**Apoptosis assay**

Apoptosis was assessed by luminescent Caspase-Glo^®^ 3/7 Assay (Promega) or by flow cytometry using annexin V (FITC) and propidium iodide (Thermo Fisher Scientific) double staining. For the luminescent assay, PASMCs were seeded in a 96-well plate at a density of 5000 cells per well. The assay was performed according to the manufacturer’s protocol 48 h post-transfection of the cells and the luminescence of the quadruplicates was measured on a luminescence microplate reader (LUMIstar Omega). For the flow cytometric analysis, PASMCs were grown on 12-well plates, transfected with the appropriate siRNAs, and starved in basal medium for 48 h, before trypsinising and harvesting the cells for annexin V (Annexin V FITC, Thermo Fisher Scientific or Annexin V APC, eBioscience) and PI staining (Thermo Fisher Scientific).

**Statistics**

Mean differences were tested using the two-sided independent-sample or the paired-sample *t*-test. The tests were performed on the logarithms of the concentrations and on the logits of proportions. One-way analysis of variance (ANOVA) with Tukey’s HSD was used to correct for multiple testing. A chi-square test of independence was performed in the case of categorical variables. Values of *p* < 0.05 were considered statistically significant. The *n* number indicates independent experiments. All graphs and statistical calculations were performed using the software package GraphPad Prism Version 5.0 (GraphPad Software, San Diego, CA, USA).
