# Supplementary material for: Long non‐coding RNAs influence the transcriptome in pulmonary arterial hypertension: the role of PAXIP1‐AS1
Source: J Pathol. 2019 Jan 16;247(3):357–70. doi: 10.1002/path.5195 (PMC6900182; doi:10.1002/path.5195)
Supplement: Supplementary file 2 — Supplementary figure legends [file PATH-247-357-s019.docx]

**Supplementary figure legends**

**Figure S1.** Enriched KEGG pathways in LCM array. (A) Summary of the barcode plots showing the individual statistics of the most significantly regulated KEGG pathways. (B) Representation of the most significant genes from the regulated KEGG pathways and representation of a minimum network analysis as performed by NetworkAnalyst (protein–protein interaction by STRING interactome with a confidence score cut-off of 900) connecting the pathways.

**Figure S2.** *PAXIP1-AS1* expression in various tissues. The expression landscape of *PAXIP1-AS1* depicted here (A, general overview; B, study-relevant selection) was obtained from the GTEx Portal on 08/01/18 and has the dbGaP accession number phs000424.v7.p2. (C) *PAXIP1-AS1* expression in selected study-relevant tissues (*n* = 1), (D) isolated cells, and (E) in donor and IPAH adventitial and parenchymal fibroblasts. *p* ≤ 0.05 as per Student’s *t*-test.

**Figure S3.** Coding potential assessment. The coding ability of the *PAXIP1-AS1* transcript was calculated by available online coding potential assessment tools (A) CPC (Coding Potential Calculator) and (B) CPAT (Coding Potential Assessment Tool).

**Figure S4.** *PAXIP1-AS1* *in situ* hybridisation. The fluorescent *in situ* hybridisation images show *PAXIP1-AS1*-stained lung tissue (A) and PASMCs (B) together with the appropriate control staining. Scale bar = 100 µm (A); 50 µm (B).

**Figure S5.** *PAXIP1-AS1* after cytokine stimulation. qRT-PCR of *PAXIP1-AS1* in PASMCs after cytokine stimulation of PASMCs for the indicated times. *p* ≤ 0.05 as per one-way ANOVA and Dunnett’s *post hoc* test.

**Figure S6.** Cross-sectional F-actin plots. Display of single cell cross-sectional analysis of F-actin fluorescence intensity signal in donor PASMCs 48 h following transfection with (A) siRNA scrambled or (B) siRNA *PAXIP1-AS1*. Single cell cross-sectional analysis of F-actin fluorescent intensity signal in (C) donor PASMCs and (D) IPAH PASMCs.

**Figure S7.** *PAXIP1-AS1* influences *FAK* expression. (A) Immunofluorescence of IPAH PASMCs 48 h after siRNA-mediated *PAXIP1-AS1* knockdown. FAK (green), F-actin (phalloidin, red), and nucleus (DAPI, blue); scale bar = 50 µm. (B) Quantification of the fluorescence intensity of FAK. AU = arbitrary units. (C) FAK levels relative to GAPDH 48 h after siRNA-mediated knockdown of *PAXIP1-AS1* in IPAH PASMCs, as determined by immunoblotting and densitometry, *n* = 5 (same samples but different blot as used in Figure 6A). *p* ≤ 0.05 as determined by Student’s *t*-test.

**Figure S8.** *PAXIP1-AS1* and *PXN* expression. (A) *PXN* gene expression in isolated PASMCs from donor and IPAH patients determined by qRT-PCR. (B) *PAXIP1-AS1* and (C) *PXN* gene expression levels determined by qRT-PCR 48 h after siRNA-mediated *PAXIP1-AS1* knockdown and co-transfection with empty or *PXN* overexpression plasmid.
