# Supplementary figures and images for: Long non‐coding RNAs influence the transcriptome in pulmonary arterial hypertension: the role of PAXIP1‐AS1
Source: J Pathol. 2019 Jan 16;247(3):357–70. doi: 10.1002/path.5195 (PMC6900182; doi:10.1002/path.5195)

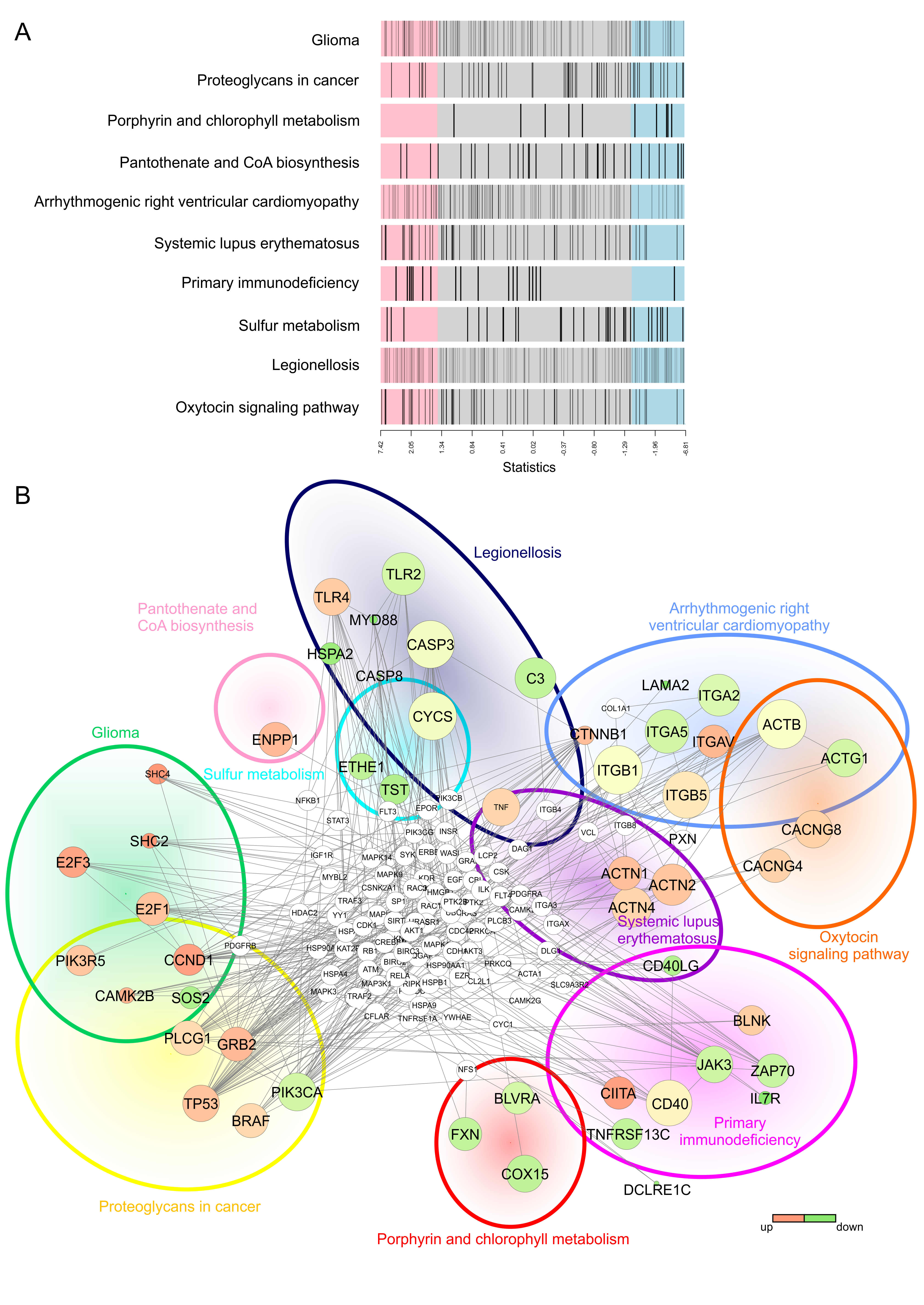

Supplement: Supplementary file 3 — Figure S1. Enriched KEGG pathways in LCM array. (A) Summary of barcode plots, showing the individual statistics of the most significantly regulated KEGG pathways. (B) Representation of most significant genes from the regulated KEGG pathways and representation of a minimum network analysis as performed by NetworkAnalyst (protein–protein interaction by STRING interactome with confidence score cut‐off of 900) connecting the pathways. [file PATH-247-357-s002.tif]

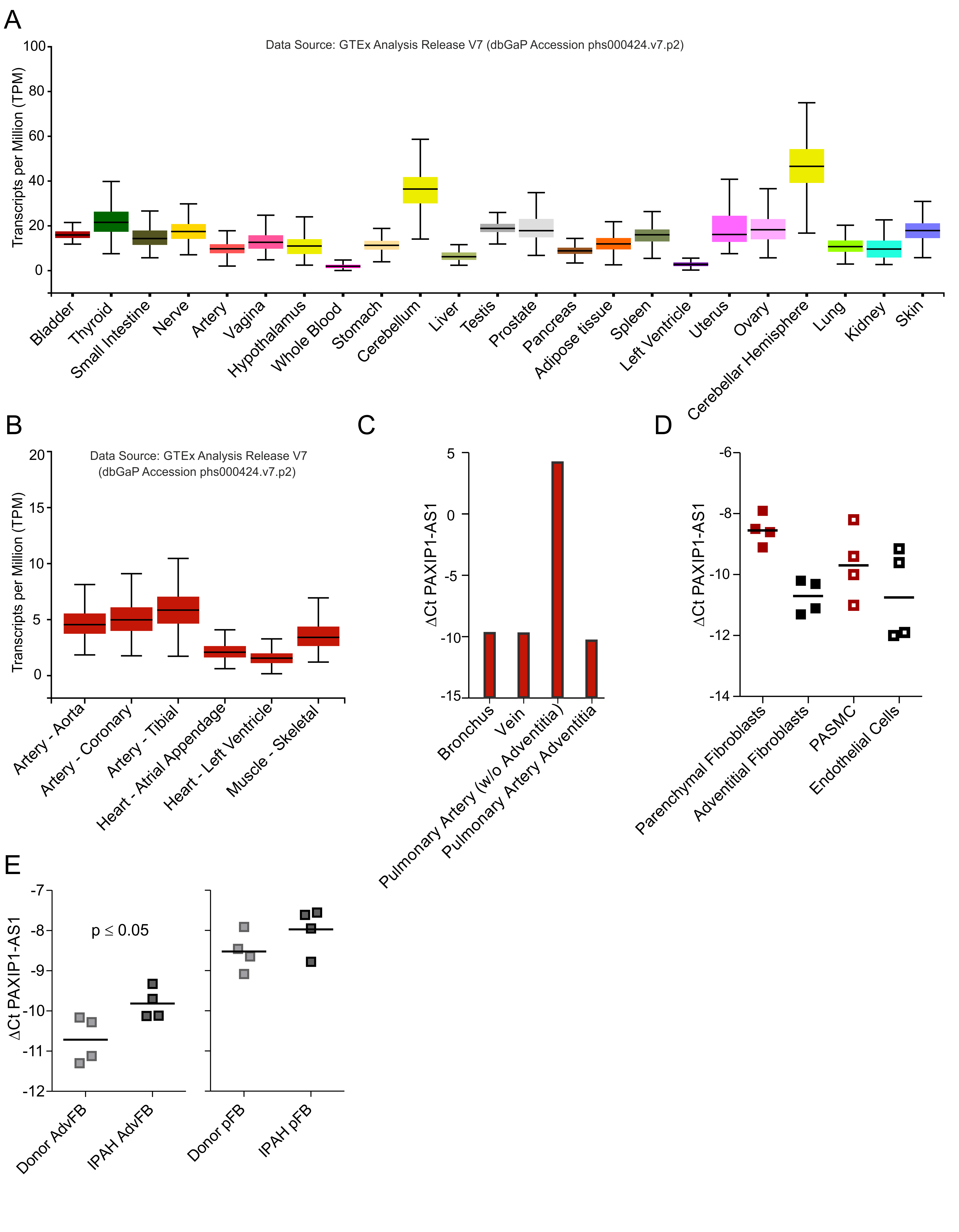

Supplement: Supplementary file 4 — Figure S2. PAXIP1‐AS1 expression in various tissues. The expression landscape of PAXIP1‐AS1 depicted here (A, general overview; B, study‐relevant selection) is obtained from the GTEx Portal on 1 August 2018 and has the dbGaP accession number phs000424.v7.p2. (C) PAXIP1‐AS1 expression in selected study‐relevant tissues (n = 1), (D) isolated cells and (E) in donor and IPAH adventitial and parenchymal fibroblasts. P ≤ 0.05 as per Student's t‐test. [file PATH-247-357-s003.tif]

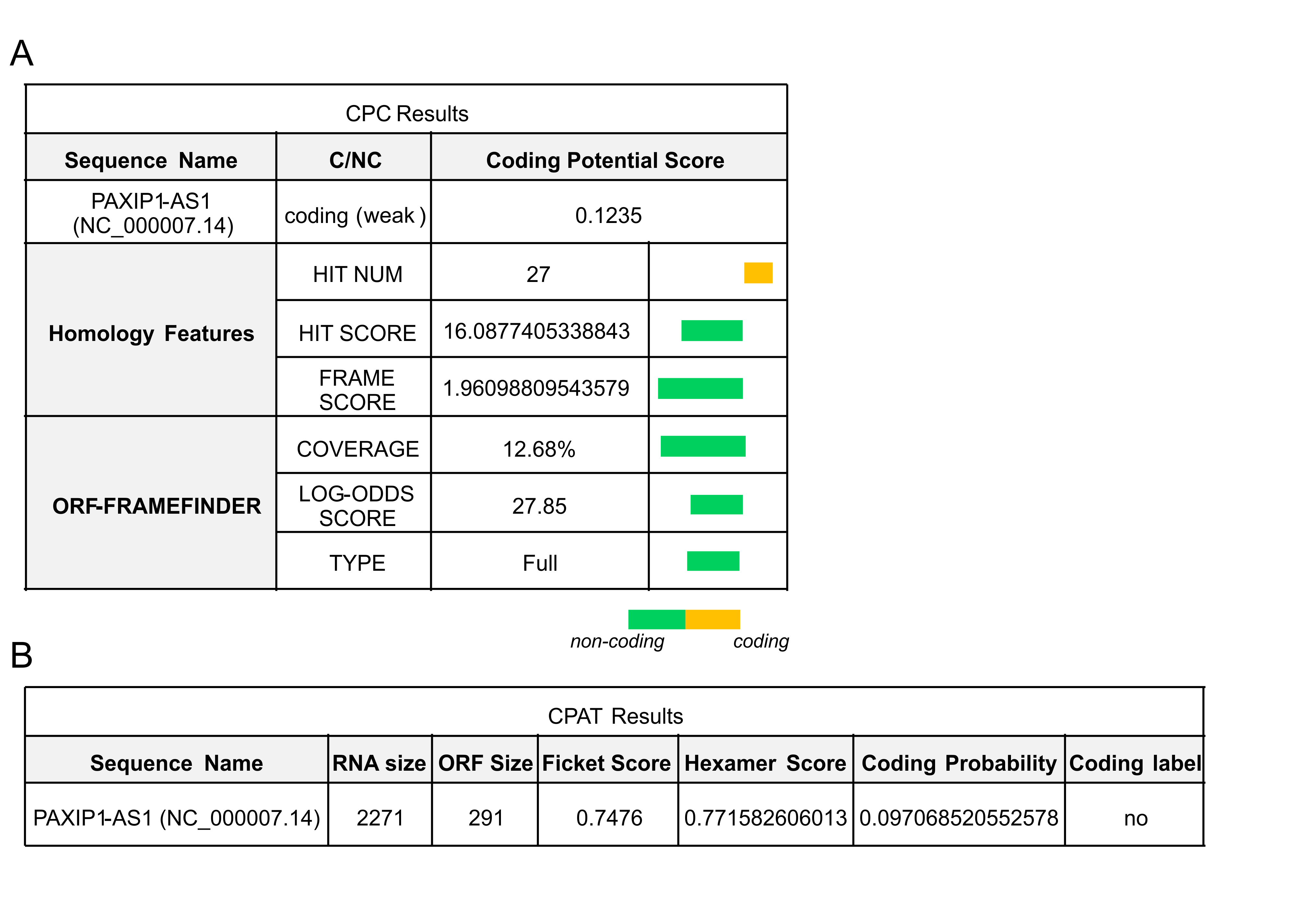

Supplement: Supplementary file 5 — Figure S3. Coding potential assessment. The coding ability of the PAXIP1‐AS1 transcript was calculated by available online coding potential assessment tools (A) CPC (Coding Potential Calculator) and (B) CPAT (Coding Potential Assessment Tool). [file PATH-247-357-s004.tif]

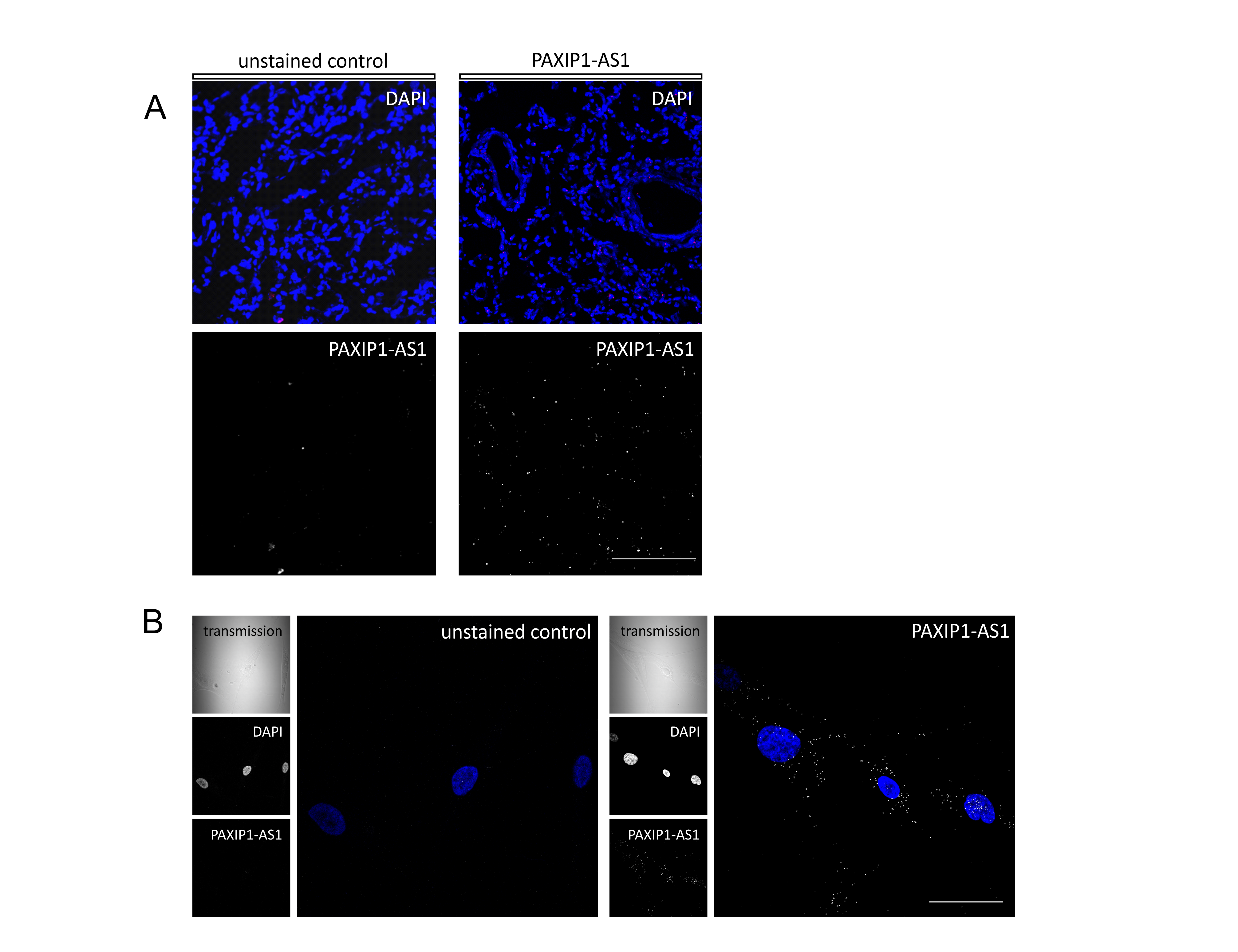

Supplement: Supplementary file 6 — Figure S4. PAXIP1‐AS1 in situ hybridization. The fluorescent in situ hybridization images show PAXIP1‐AS1‐stained lung tissue (A) and PASMC (B) together with the appropriate control stainings. Scale bar = 100 μm for (A) and 50 μm for (B). [file PATH-247-357-s005.tif]

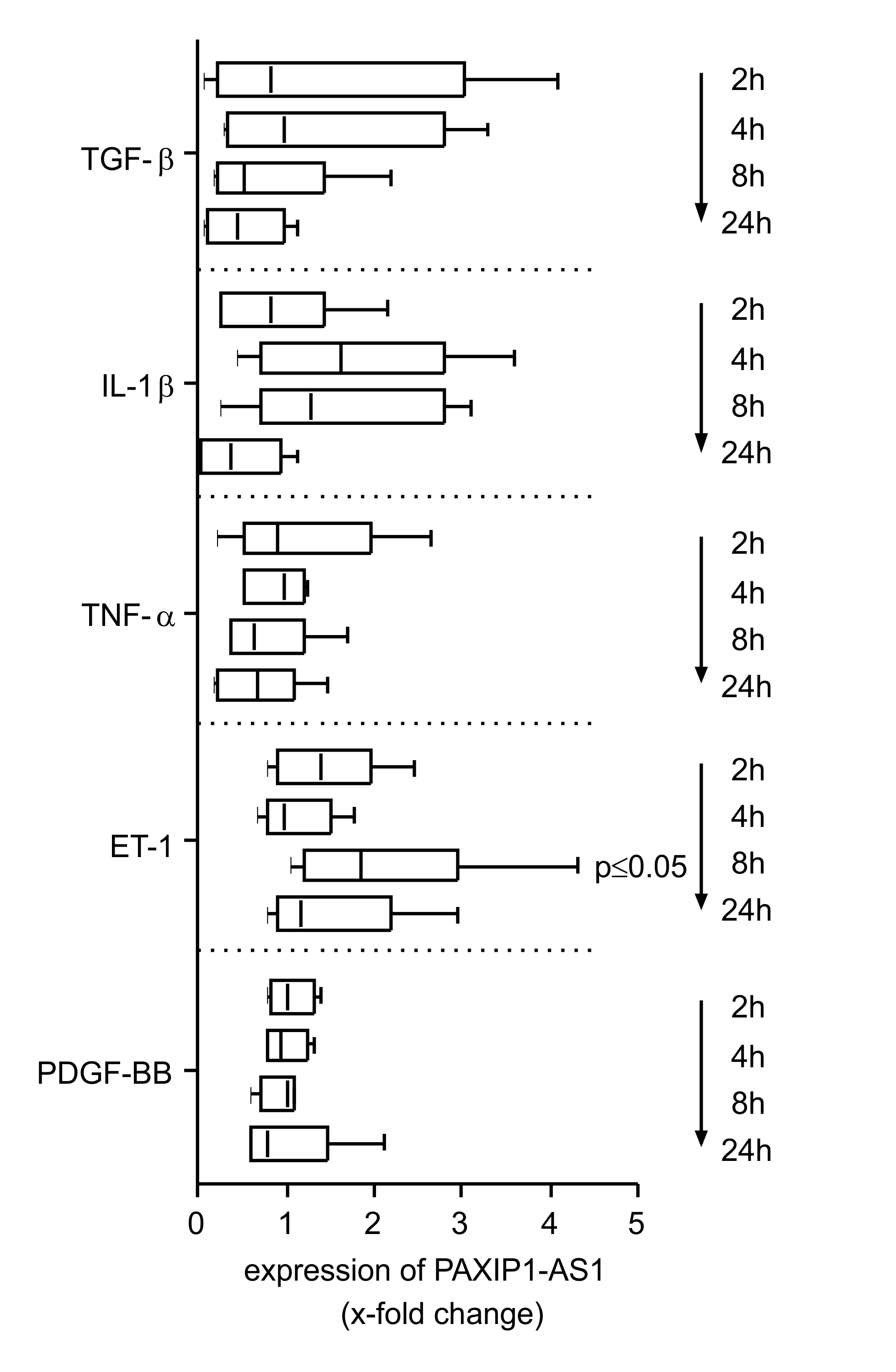

Supplement: Supplementary file 7 — Figure S5. PAXIP1‐AS1 after cytokine stimulation. qRT‐PCR of PAXIP1‐AS1 in PASMC after cytokine stimulation of PASMC for indicated times. P ≤ 0.05 as per one‐way ANOVA and Dunnett's post hoc test. [file PATH-247-357-s006.tif]

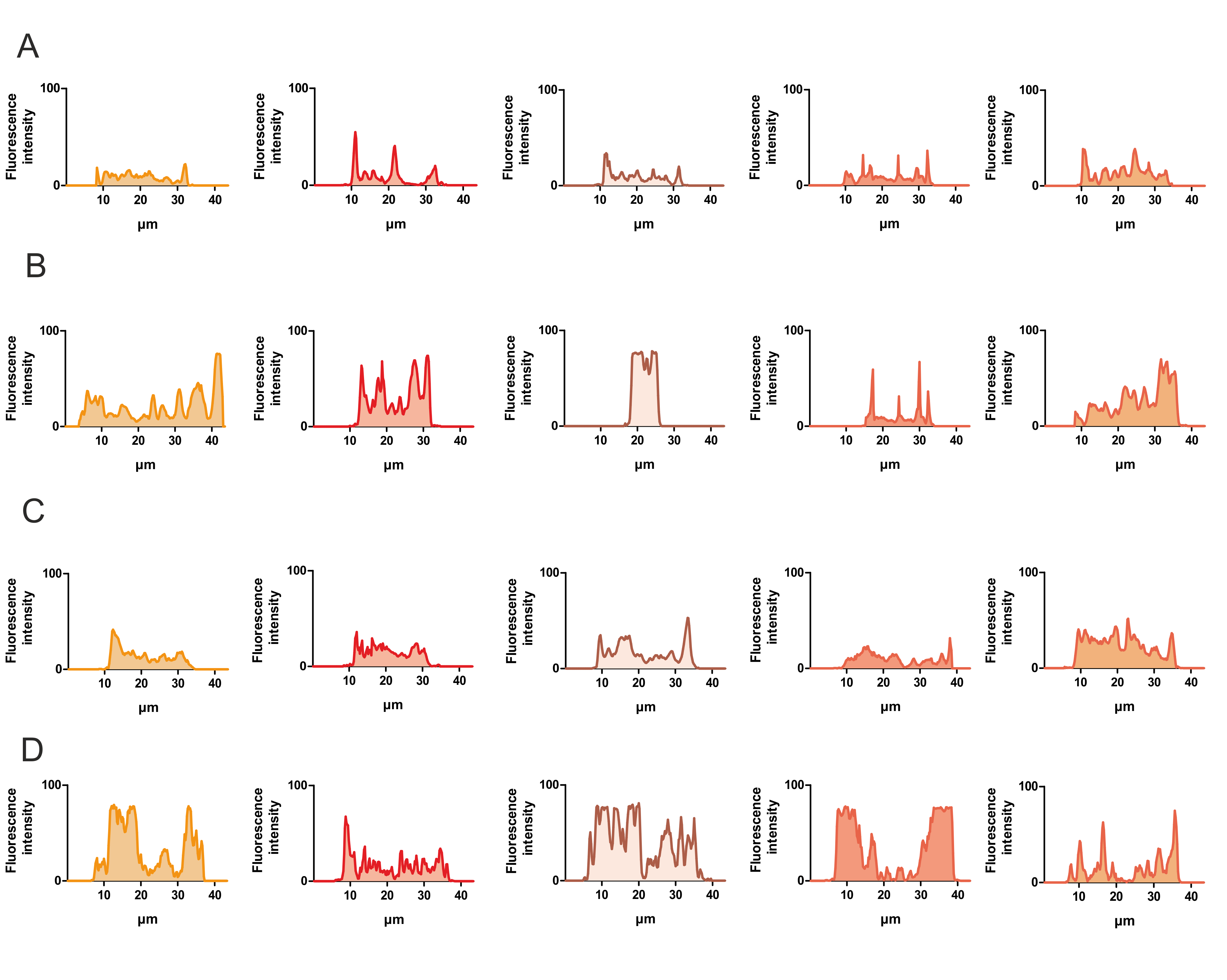

Supplement: Supplementary file 8 — Figure S6. Cross‐sectional F‐actin plots. Display of single cell cross‐sectional analysis of F‐actin fluorescence intensity signal, in donor PASMC 48 h following transfection with (A) siRNA scrambled or (B) siRNA PAXIP1‐AS1. Single cell cross‐sectional analysis of F‐actin fluorescent intensity signal in (C) donor PASMC and (D) IPAH PASMC. [file PATH-247-357-s007.tif]

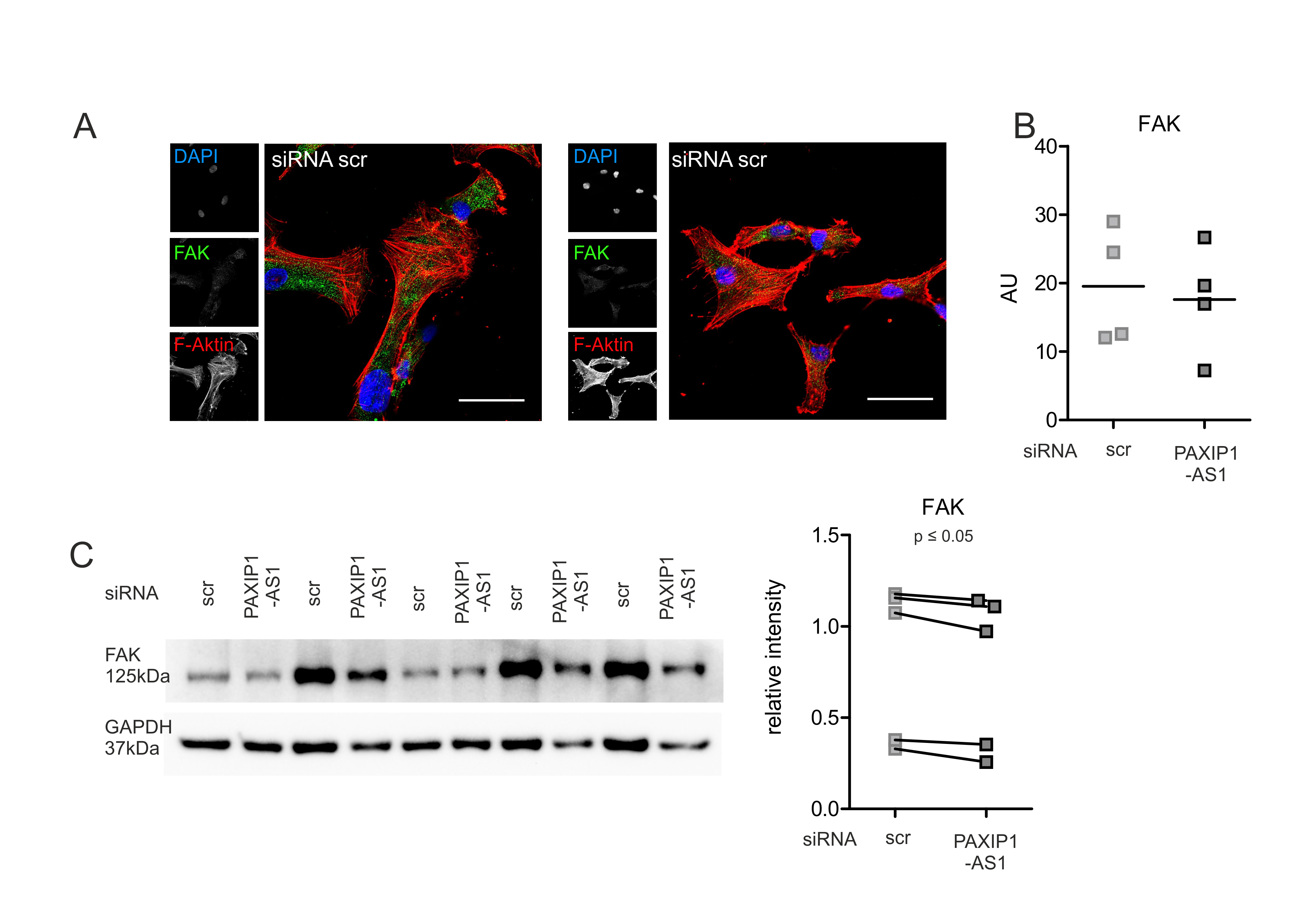

Supplement: Supplementary file 9 — Figure S7. PAXIP1‐AS1 influences FAK expression. (A) Immunofluorescence of IPAH PASMC 48 h after siRNA‐mediated PAXIP1‐AS1 knockdown; FAK (green), F‐actin (phalloidin, red) and nucleus (DAPI, blue); scale bar = 50 μm. (B) Quantification of fluorescence intensity of FAK; AU = arbitrary units. (C) FAK levels relative to GAPDH 48 h after siRNA‐mediated knockdown of PAXIP1‐AS1 in IPAH PASMC, as determined by immunoblotting and densitometry, n = 5 (same samples but different blot as used in Figure 6A). P ≤ 0.05 as determined by Student's t‐test. [file PATH-247-357-s008.tif]

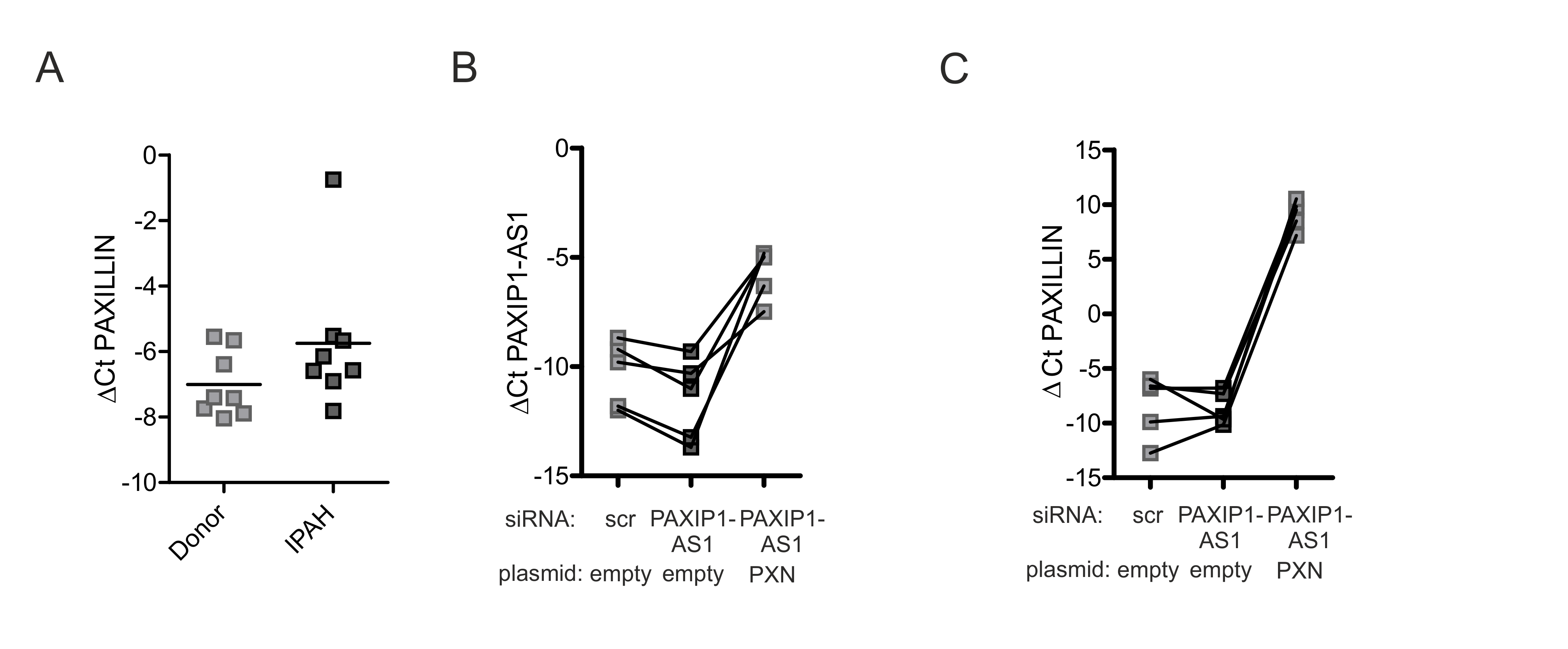

Supplement: Supplementary file 10 — Figure S8. PAXIP1‐AS1 and PXN expression. (A) PXN gene expression in isolated PASMC from donor and IPAH patients, determined by qRT‐PCR. (B) PAXIP1‐AS1 and (C) PXN gene expression levels, determined by qRT‐PCR 48 h after siRNA‐mediated PAXIP1‐AS1 knockdown and co‐transfection with empty or PXN overexpression plasmid. [file PATH-247-357-s009.tif]
