# Supplementary material for: Long non‐coding RNAs influence the transcriptome in pulmonary arterial hypertension: the role of PAXIP1‐AS1
Source: J Pathol. 2019 Jan 16;247(3):357–70. doi: 10.1002/path.5195 (PMC6900182; doi:10.1002/path.5195)
Supplement: Supplementary file 19 — Table S9. Primer sequences [file PATH-247-357-s018.docx]

**Table S9.** Primer sequences

| **Gene name** | **Forward (5′–3′)** | **Reverse (5′–3′)** |
| --- | --- | --- |
| *PAXIP1-AS1* | ACAAACCGGAGTCCAGGAAC | CAATGCTCCACAGTTTTACGAC |
| *PXN* | TGGACAGCCCTACTGTGAAA | AGAAGTGTTCAGGGTGCCA |
| *PTK2* | GAGTCCAGAAGACAGGCCAC | CCAGGGTAGCCAGAAACCTG |
| *ACTB* | TCAAGATCATTGCTCCTCCTGAG | TCCTGCTTGCTGATCCACATC |
| *B2M* | CCTGGAGGCTATCCAGCGTACTCC | TGTCGGATGGATGAAACCCAGACA |
| *LINC01398* | CCTCGGTTTCTGAGGGTCTG | ACCATGGAGGGTTTGGTTGG |
| *TUSC8* | ACTGGCCTTCCCAGAAACAG | GTTCACCTCCACCGGAAGG |
| *LINC00877* | CGAGAAGGAAAGCCGGTGAT | TCCATGTGTCTGCTTTGCCT |
| *JARID1-AS1* | CCTCAACGAGGTCCGGAATG | CAGAGGGCTGGGTAGAATGC |
| *SNHG16* | AGTAATCGCCATGCGTTCTTTG | GGTTTTCCAGAATAATCTCAGTTG |
| *LINC01214* | CAGAGGGACTTTCAGTTTTTCAC | CAAGTGCCCAAGTAACAAAAGG |
